# Biphasic Solar Reforming of Lignin to Aromatics and CO for In Situ Carbonylation of Organics

**DOI:** 10.1021/jacs.6c10525

**Published:** 2026-08-28

**Authors:** Diksha Mittal, Sampurna Mitra, Etienne Bonvin, David R. Spring, Shannon A. Bonke, Erwin Reisner

**Affiliations:** Yusuf Hamied Department of Chemistry, 2152 University of Cambridge, Lensfield Rd, Cambridge CB2 1EW, U.K.

## Abstract

Lignin is a large-scale, renewable carbon source that remains underutilized due to its complex polymeric structure. Photocatalytic conversion of lignin promises selective bond cleavage to yield functionalized products but is hindered by its strong light absorption. Herein, we report a biphasic photocatalytic system that avoids light competition and enables the generation of both CO and functionalized aromatics from Kraft lignin under visible light at ambient temperature and pressure. This system uses water-dispersed CdS quantum dots to photocatalyze the conversion of Kraft lignin’s aldehyde and hydroxyl groups to CO via a decarbonylation mechanism. The biphasic system enables this biomass-derived CO to be used in situ for aminocarbonylation. Concurrent cleavage of the β-O-4 bonds in Kraft lignin releases functionalized aromatic products with 81% yield. This work demonstrates the simultaneous light-driven production of sustainable aromatics and one-pot utilization of lignin for carbonylation of organics via cascade reactions.

## Graphical abstract

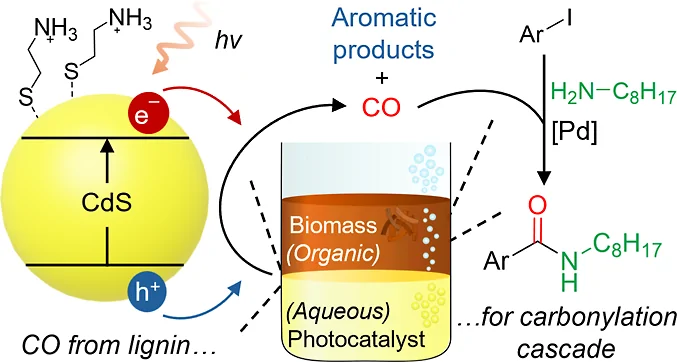

## Introduction

Production of cellulose pulp from nonedible lignocellulose biomass generates 50–70 Mt of lignin annually (15–30 wt.% of lignocellulose).^1,2^ This lignin is largely discarded as waste or burned for power, which emits CO_2_. In the petrochemical sector, CO and aromatic compounds are primarily fossil-derived by using carbon-intensive processes. However, the catalytic valorization of lignin under mild conditions would render it a large-scale sustainable source of carbon feedstocks and aromatic compounds.^3^

Extracting useful chemicals from lignin is challenging and established approaches often use high temperatures and pressures. The generation of CO from biomass uses gasification or pyrolysis at high temperatures (>750 °C), but this does not release the most valuable aromatic products,^4,5^ and there are very limited examples at milder conditions.^6^ To extract aromatic products, lignin can be activated by classic Brønsted or Lewis acids for hydrolysis, but this approach lacks selectivity and results in significant repolymerization.^7,8^ Hydrogenolysis enables more selective release of aromatic products, but not CO, and it relies on platinum group metal catalysts under high pressure and moderate temperature (20–60 bar, 150–200 °C).^9–11^ Consequently, new strategies that selectively and energy-efficiently valorize lignin into small aromatics and carbon feedstocks are required to utilize it as a sustainable resource.

Photocatalytic reactions can proceed at mild conditions through redox mechanisms with multiple lignin functionalities, such as oxidative activation of C–O bonds. The β-O-4 ether motifs that link 30–50% of the lignin structure together (depending on wood type^12^) can be cleaved to extract small aromatic molecules. The photocatalytic cleavage of molecular model compounds containing β-O-4 or similar motifs has been shown using semiconductors, such as C_3_N_4_,^13–15^ CdS,^16–20^ ZnIn_2_S_4_,^21–24^ and TiO_2_.^25–27^ However, polymeric lignin is a more complex and challenging substrate than the model compounds, given the intertwining of aryl-ether (β-O-4) bonds in lignin with other motifs, including resinol (β–β), phenylcoumaran (β-5), and dibenzodioxocin, among others (Figure 1). Among the few examples of β-O-4 cleavage in lignin, pioneering work using CdS quantum dots has shown release of aromatic monomers from lignin via redox pathways.^20^

**Figure 1:**
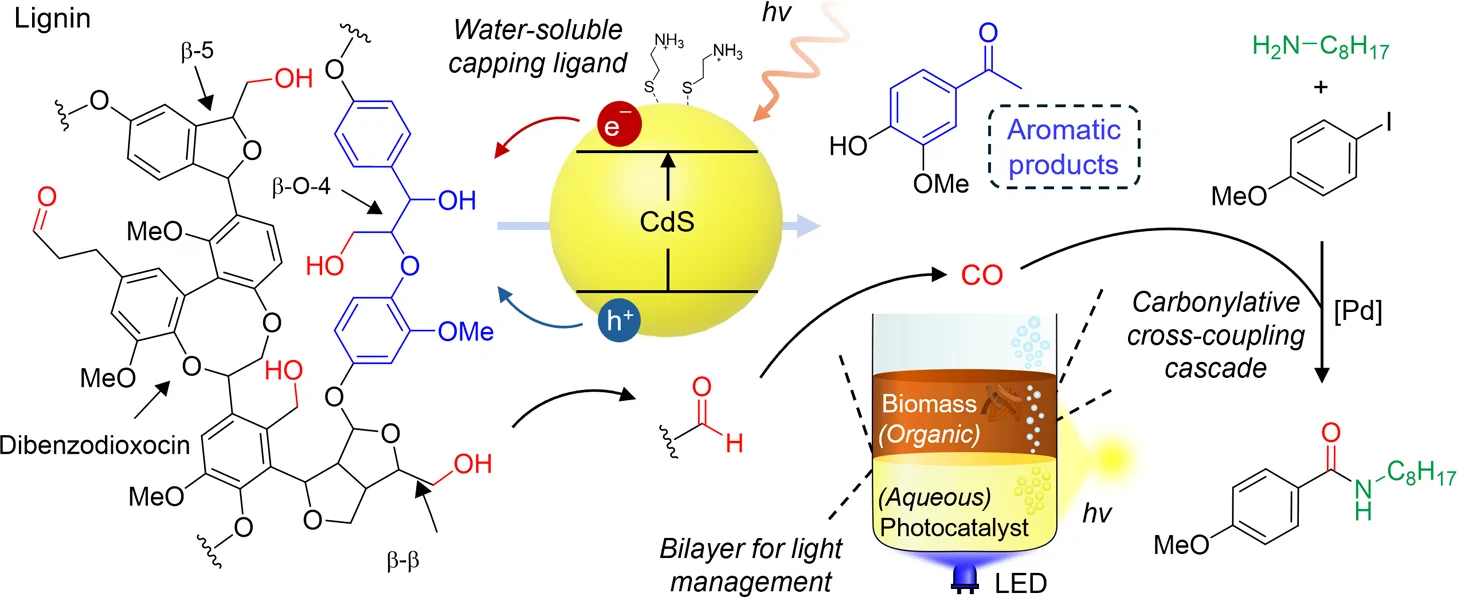
Schematics of photoreforming lignin to CO and cleavage of the dominant β-O-4 motif over other lignin linkages (β–β, β-5, and dibenzodioxocin, among others) to yield functionalized aromatic monomers using a CdS quantum dots (QDs) photocatalyst in a biphasic scheme under LED or simulated solar light irradiation. The biomass-derived CO is further utilized in a one-pot cascade reaction for carbonylative cross-coupling.

Key limitations in photocatalytic lignin cleavage include poor light penetration due to the use of bulk, solid semiconductors and strong visible-light absorption by lignin. There are also no photocatalytic systems that generate gaseous carbon intermediates, such as CO, from lignin, which would be beneficial due to the easy separation of residual lignin from gases. Generating CO from lignin also provides a handle for developing solid CO surrogates for downstream utilization. It bypasses the challenges associated with photocatalytic CO_2_ reduction to CO for such applications, which typically rely on concentrated CO_2_ sources and sacrificial electron donors.^28–30^ Thus, photocatalytic systems that avoid light competition and can valorize lignin to generate a clean, readily utilizable carbon source, as well as functionalized aromatics, are needed to make the process more effective and energy efficient.

In this work, we present a biphasic system for the photocatalytic conversion of lignin to both CO and functionalized aromatics at ambient temperature and pressure under LED or simulated solar light irradiation (Figure 1). The biphasic system prevents light competition between lignin and the catalyst, which is achieved by compartmentalizing a fully water-dispersible thiol-capped CdS quantum dot (QD) catalyst in the aqueous bottom layer while lignin is in an immiscible organic layer where it has higher solubility (top layer) in a bottom or side irradiation configuration. This compartmentalization also facilitates the postreaction separation of the catalyst from the substrates and the lignin-derived aromatic monomers. In situ utilization of biomass-derived CO in a carbonylative cross-coupling reaction is shown by exploiting the biphasic solvent system to establish a one-pot cascade reaction, demonstrating the potential to replace external handling of toxic and fossil-derived CO. This system also cleaves the β-O-4 bonds of polymeric Kraft lignin and basic and complex model compounds to generate aromatic products. The mechanistic pathway for CO generation was determined to involve terminal lignin hydroxy and aldehyde groups, facilitated by a cocatalytic thiol ligand. Sourcing aromatic products and CO from lignin and utilizing the CO in situ for organic synthesis under visible light opens a new avenue for higher lignin utilization.

## Results and Discussion

### Bilayer Assembly

The light absorption of lignin extends into the visible region and competes with the photocatalyst’s absorption, thereby hindering photocatalytic performance (Figure S1). This limitation was addressed herein by developing a biphasic configuration, specifically, a photocatalyst in the aqueous layer and lignin in the immiscible organic layer. The aqueous layer required a photocatalyst system that could oxidize the β-O-4 ether bonds of lignin while being optically transparent and dispersible in water. These criteria are fulfilled by CdS quantum dots, which are inexpensive and absorb visible light (bulk bandgap of 2.4 eV), with valence and conduction band energies suitable for β-O-4 bond cleavage as required for lignin valorization (1.7 V and −0.7 V vs the standard hydrogen electrode, SHE, respectively).^20,31,32^

The dispersibility of the QDs can be controlled by exchanging the capping ligands. Thus, the hydrophobic oleic acid ligand from QD preparation was exchanged with hydrophilic 2-aminoethanethiol ligand to transfer the CdS QDs from apolar organic to aqueous media.^33,34^ Characterization of the CdS QDs with oleic acid (CdS-OA) and 2-aminoethanethiol (CdS-AET) ligands using UV–vis absorption spectroscopy, transmission electron microscopy, and X-ray photoelectron spectroscopy indicated full dispersibility of the QDs in water with no aggregation, alteration in size (5.9 ± 0.9 vs 5.6 ± 0.7 nm) or electronic structure change (first excitonic peak at 462 nm), along with no surface oxidation of CdS-AET QDs upon ligand exchange (characterization in Supporting Information Note S1 and Figures S2–S5).

Ethyl acetate (EtOAc) was chosen as the water-immiscible lignin-containing layer as it is a biodegradable and industry-preferred solvent.^35^ Kraft lignin, an abundant byproduct of lignocellulose processing in the paper pulp industry, was used as a substrate with direct industrial relevance. Upon refluxing in EtOAc for 1 h, the insoluble material was filtered to give a saturated solution containing 3.3 mg mL^–1^ of lignin.

### Photocatalytic CO Generation from Lignin

To assess the bilayer system efficacy for lignin valorization, the Kraft lignin-saturated EtOAc solution was layered onto the aqueous CdS-AET QD solution to assemble the bilayer (Figure 2a inset). The aqueous layer contained CdS-AET in water (1 μM, 0.333 mg mL^–1^), which showed an absorbance of 1.5 AU at the illumination wavelength of 447 nm for high light utilization. The EtOAc contained Kraft lignin (3.3 mg mL^–1^) with an absorbance of 6.5 AU, and the system had a 1:1 EtOAc/H_2_O ratio (3 mL total volume). The samples were placed under an inert atmosphere (N_2_) and illuminated from below by an LED (447 nm, 120 mW cm^–2^) for 24 h at 25 °C, with orbital convection (250 rpm), prior to product identification and quantification by gas chromatography–mass spectrometry (GC–MS) and high-performance liquid chromatography (HPLC).

**Figure 2:**
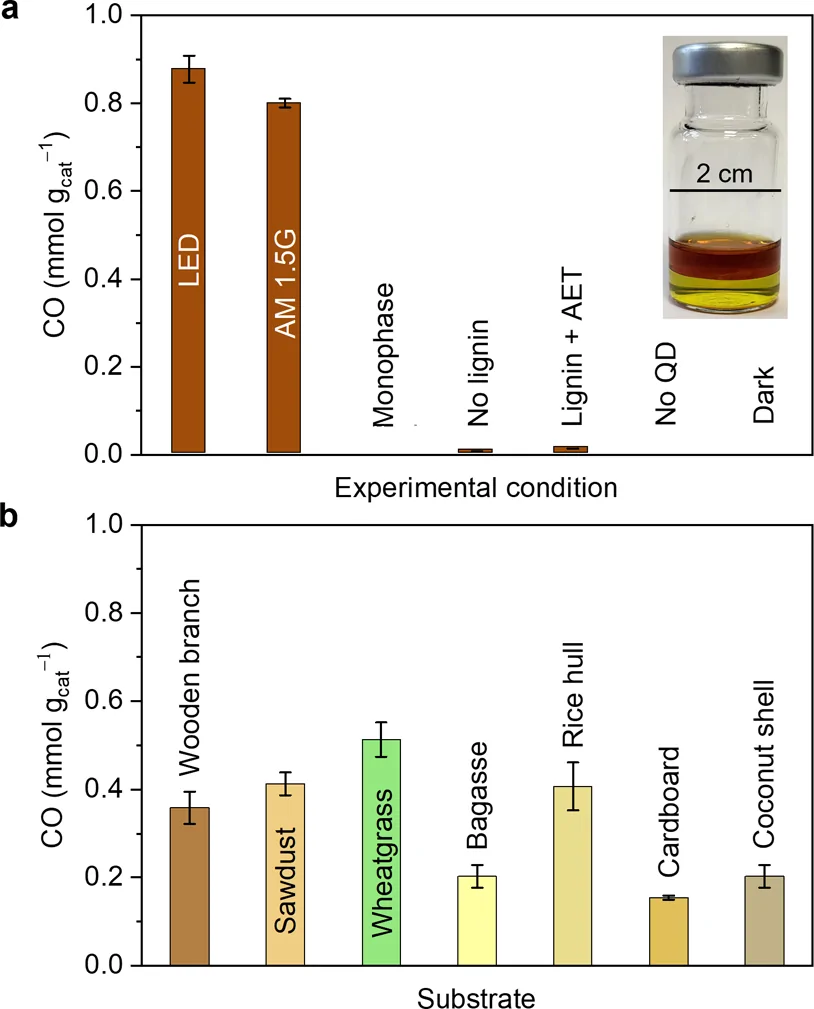
CO generation through photoreforming lignin on CdS-AET QDs. Photocatalytic CO production (447 nm LED, 120 mW cm^–2^ or simulated solar light AM 1.5G, 100 mW cm^–2^) with CdS-AET QDs (1 μM) in 3 mL of H_2_O/EtOAc (1:1) from 3.3 mg mL^–1^ Kraft lignin along with exclusion controls (a) and raw lignin substrates (b). Exclusion controls and experiments with raw lignin substrates were conducted under LED illumination (447 nm, 120 mW cm^–2^). Monophase control was performed using a 2:1 H_2_O/MeCN (3 mL) solvent mixture. The inset photograph shows the bilayer with the aqueous CdS-AET QDs at the bottom and the EtOAc lignin layer above. All measurements were performed at 25 °C under a N_2_ atmosphere with 250 rpm orbital shaking for 24 h, in triplicate, and errors are given as ± one standard deviation.

In the biphasic system, CdS-AET QDs access lignin at the liquid–liquid interface with orbital convection enhancing the interfacial area to maximize contact. Upon illumination, the system generated 0.88 ± 0.03 mmol_CO_ g_cat_^–1^ after 24 h, thereby demonstrating that lignin can serve as a source of CO under mild photocatalytic conditions (Figure 2a and Table S1). Examining the reaction kinetics revealed a CO generation rate of 0.96 mmol_CO_ g_cat_^–1^ h^–1^ within the first hour before plateauing by 2 h (Figure S6). The reaction was run for 24 h, however, to allow sufficient time for β-O-4 bond cleavage in lignin and consequent generation of aromatic monomers in the liquid phase (see below). Neither H_2_ nor formate was detected, while CO_2_ was present at <0.5% of the CO amount, indicating that >99.5% pure CO was generated in N_2_. All components, including the CdS-AET QDs, Kraft lignin, and light, were identified as essential to CO generation using exclusion controls. Control measurements without the substrate did not result in any CO generation, indicating the stability of EtOAc and CdS-AET QDs under the reaction conditions (Table S1). CO formation was at trace levels when CdS QDs were excluded, i.e., the AET ligand was added with Kraft lignin-saturated EtOAc, ruling out the possibility of lignin acting as the photocatalyst. Experiments substituting LED illumination with simulated solar light (AM 1.5G, 100 mW cm^–2^; side illumination) yielded 0.80 ± 0.01 mmol_CO_ g_cat_^–1^, confirming that the reaction is viable under solar irradiation. This photocatalytic generation of CO from lignin has not been previously described, but offers the potential to use lignin as a clean feedstock for further chemical utilization without purification or isolation from the substrate.

To demonstrate the importance of the bilayer in preventing lignin from blocking light from reaching the photocatalyst, a control experiment was conducted using a 2:1 H_2_O/MeCN (3 mL) solvent mixture to disperse the CdS QDs and lignin at the same loadings in a miscible solvent. In this control, no lignin conversion or CO evolution was obtained from the reaction, thereby confirming the importance of the bilayer for light management (Figure 2a). The use of a bilayer also limits substrate–catalyst interaction to the interface, although emulsification of the interface with orbital shaking increases the interfacial reaction area (Figure S7). Limitations imposed by the bilayer were probed by varying the orbital convection rate, which revealed that an increase yielded a minor enhancement in the reaction rate, whereas a decrease significantly lowered the rate (0.36, 0.96, and 1.04 mmol_CO_ g_cat_^–1^ h^–1^ at 50, 250, and 400 rpm, respectively; Figure S6). The reaction was thus neither in a convection-limited regime, nor could the reaction rate be significantly increased by increasing the interfacial area via the convection rate.

Unprocessed lignocellulose was then examined for CO generation to show the broad applicability of this reaction to biomass conversion beyond Kraft lignin. The raw lignocellulosic biomass waste samples were ground and refluxed in EtOAc for 1 h to extract lignin, which was reacted in a biphasic configuration using the experimental parameters defined for Kraft lignin. The raw biomass tests showed CO evolution from wood, sawdust, wheatgrass, bagasse, rice hull, cardboard, and coconut shell, confirming that the observed reaction is broadly applicable to lignin. The highest CO yield was from wheatgrass, followed by sawdust, rice hull and tree branch (0.51 ± 0.04, 0.41 ± 0.03, 0.41 ± 0.05 and 0.36 ± 0.04 mmol_CO_ g_cat_^–1^; Figure 2b and Table S1). A low CO yield was observed with cardboard (0.15 ± 0.01 mmol_CO_ g_cat_^–1^), which has a low lignin content. In all cases, these raw substrates yielded lower CO than Kraft lignin (0.88 ± 0.03 mmol_CO_ g_cat_^–1^). Control experiments using dissolved cellulose and hemicellulose showed negligible contribution toward CO evolution (0.061 ± 0.001 and 0.07 ± 0.01 mmol_CO_ g_cat_^–1^, respectively; Table S1), and the presence of a significant polysaccharide (lignin-carbohydrate complex) component in the raw samples lowers the lignin availability and results in no correlation between the amount of dissolved material and CO yield. Thus, Kraft lignin is the highest-performing substrate and the most useful as it is a large-scale industrial waste product. The system reported herein also demonstrates broad applicability, including the use of raw biomass. It thereby enables the use of ubiquitous waste biomass as a source of both functionalized aromatic monomers and CO under visible light illumination.

### Carbonylation Cascade Using CO from Lignin

CO utilization reactions conventionally rely on energy-intensive methods, such as steam reforming of methane for CO generation, which introduces logistical and safety challenges. Carbonylation reactions can be carried out using CO generated by photocatalytic CO_2_ reduction, which offers a renewable carbon source.^28,29,36^ However, the photocatalytic activation and reduction of CO_2_ to CO typically require concentrated CO_2_ feeds and sacrificial electron donors.

The CO evolved from Kraft lignin is biomass-derived and provides a handle to use biomass as the CO source for sustainable organic synthesis. To this end, the biphasic solvent system used herein for light management was exploited to enable a one-pot compartmentalized cascade reaction,^37^ whereby CO was generated from lignin at the interface and utilized for carbonylation in the organic phase containing lignin. Aminocarbonylation was selected for implementation in the cascade, as it is a fundamental and widely used cross-coupling reaction that employs catalytic palladium catalysts to couple an aryl halide with CO and an amine to form an aryl amide in an organic solvent and proceeds at room temperature.^38^

The biphasic system was prepared as previously (1 μM CdS-AET QDs in H_2_O, sat. lignin in EtOAc under N_2_), with excess carbonylation reagents (0.1 mmol *p*-iodoanisole and 0.5 mmol octylamine) and a cross-coupling catalyst (20 mol% Pd­(OAc)_2_ with 40 mol% Xantphos ligand) added into the EtOAc layer (conditions based on our prior work),^37^ with 1 equiv. CO obtained from lignin under standard photocatalytic conditions. Illumination of this biphasic system resulted in a cascade reaction that formed 4-methoxy-*N*-octylbenzamide with a 68% yield under CO-limited conditions (Table 1, Entry 1; Table S2 and Figure S8). No external CO was added to the reaction, meaning that all CO used was derived from lignin in situ. CO generation is ca. 90% complete within 2 h, at which point the carbonylation yield is 62% (i.e., ca. 90% of the 24 h yield), indicating that the CO generation and utilization rates are matched. Fittingly, the rate of CO generation of 0.96 mmol_CO_ g_cat_^–1^ h^–1^ (Figure S6) corresponds to 0.48 μmol h^–1^, which is similar to the carbonylation rate for such a system (≳0.4 μmol h^–1^).^37^ Increasing the Pd loading 5-fold to stoichiometric levels only increased the carbonylation yield by 9% (68–77%), indicating that the carbonylation rate is broadly matched/limited by the CO generation rate and leading to effective utilization. Testing the cascade reaction using simulated solar light (AM 1.5G, 100 mW cm^–2^) rather than LEDs resulted in 48% yield of 4-methoxy-*N*-octylbenzamide (Table 1, Entry 2), confirming the suitability of solar light to initiate the reaction cascade.

**Table 1:**
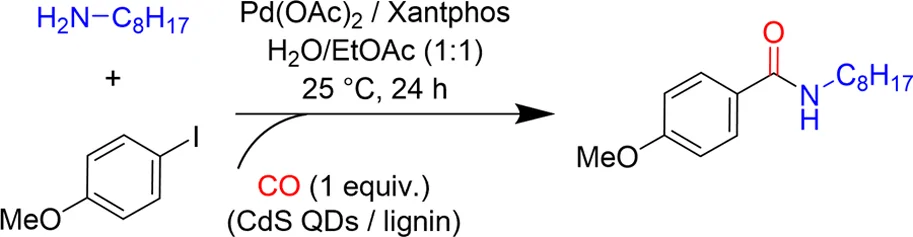
One-Pot Cascade Reaction Utilizing Kraft Lignin as a Green CO Source for Aminocarbonylation^a^ ^,^ ^b^

entry	system	yield (%)
**1**	LEDs	68
**2**	solar light	48
**3**	no QDs	n.d.
**4**	no [Pd]	n.d.
**5**	no lignin	n.d.
**6**	no light	n.d.
**7**	no biphase^c^	n.d.
**8**	two-compartments^d^	3

a Biphasic solvent system (1:1, 3 mL) under a N_2_ atmosphere with aqueous layer containing CdS-AET QDs (1 μM) and EtOAc layer containing sat. Kraft lignin (3.3 mg mL^–1^), iodoanisole (0.1 mmol), octylamine (0.5 mmol), Pd­(OAc)_2_ (20 mol %) and Xantphos (40 mol %) at 25 °C with 250 rpm orbital shaking for 24 h. LED illumination at 447 nm (120 mW cm^–2^) unless specified as simulated solar light (AM 1.5G, 100 mW cm^–2^). Yields determined by GC–MS.

b The yields were recorded under CO-limited conditions.

c 2:1 H_2_O/MeCN (3 mL) solvent system.

d Two-compartment cell with one pot for CO generation from lignin and one pot for carbonylation joined by a shared headspace of smaller volume than the biphasic cascade (2.5 vs 8.7 mL). n.d. = not detectable.

The generation of aromatic monomers from lignin was measured to not be affected by the carbonylation cascade, indicating that the carbonylation reagents and lignin cleavage products did not interfere with each other, i.e., compatibility of the Pd/Xantphos cross-coupling catalyst with lignin and the aromatic monomers. Pd catalysts are known to be sensitive to thiols; however, the biphasic configuration keeps the hydrophilic thiol ligand separated from the organosoluble Pd/Xantphos catalyst to enable the carbonylation cascade. Exclusion controls confirmed that the CdS-AET, Pd-catalyst, lignin, and light were required to obtain the aminocarbonylation product (Table 1, entries 3–6), consistent with all of CdS-AET, lignin, and light being required for CO generation from lignin. A further control experiment using a miscible 2:1 H_2_O/MeCN solvent system did not yield any product, as lignin prevents light reaching the QD photocatalyst and thereby prevents the generation of CO (Table 1, entry 7). Separating the carbonylation into two compartments, one for CO generation from lignin and one for carbonylation (Figure S9), as shown in the literature,^30^ yielded only 3% product despite the headspace being smaller than that used for the cascade (Table 1, entry 8).

The higher yield in the bilayer cascade is attributed to CO generation at the interface, resulting in a high local CO concentration in the EtOAc layer, combined with carbonylation proceeding at a rate matching (or exceeding) that of CO generation. This combination enables the CO to be utilized for carbonylation before it equilibrates in the headspace. In contrast, the two-compartment system requires CO to first equilibrate into the bulk headspace and then into the carbonylation solvent, which limits the system by the low dissolved CO concentration. Partitioning of CO into the headspace prevents its concentration in the carbonylation solvent from exceeding the equilibrium level at any point, which severely hinders the carbonylation reaction and results in a trace yield.

A photocatalytic cascade that uses Kraft lignin as a CO source for carbonylation was demonstrated. The cascade replaces toxic, typically fossil-derived CO gas with waste-biomass-derived CO in organic synthesis, enabling a one-pot process.

### Lignin β-O-4 Linkage Cleavage to Generate Aromatics

In addition to the CO, functionalized aromatic monomers from cleavage of the β-O-4 bonds in lignin are also generated from the reaction.^20^ The total yield of all monomers quantified from the reaction was 19 wt.% after 24 h. The highest yielding products were vanillin, guaiacol, and derivatives thereof with propanol and hydroxy ketone groups (6.2, 5.0, 3.6, and 2.8 wt.% of the starting lignin mass, respectively; Table S3 and Figure S10). The maximum yield of monomers that can be extracted via β-O-4 bond cleavage in lignin was determined to be 23 wt.% using heteronuclear single quantum coherence (HSQC) NMR spectroscopy (Figure S11 and Table S4; following established procedure^20^). Therefore, the system results in an excellent 81% yield of valuable aromatic monomers. The unreleased monomers are likely connected to the complex lignin structure via partial connections to other linking motifs, such as resinol and phenylcoumaran, which prevent their release via β-O-4 cleavage. As the first report of simultaneous generation of CO and aromatics from lignin, this catalytic system demonstrates state-of-the-art photocatalytic conversion of lignin, where reports on photocatalytic valorization of polymeric lignin are rare and inefficient due to poor light penetration.^13,16,18,20,21^

The system stability and isolation of these aromatic products from crude reaction mixture was then examined. The CdS QDs in the aqueous phase were reused over ten consecutive reaction cycles, with fresh Kraft lignin-saturated EtOAc solution added each time, and displayed no loss of monomer yield, e.g., vanillin yield of 5.8 ± 0.3 wt.% over 10 cycles (Table S5; CO yield discussed in mechanism section). Following all reactions, the EtOAc layers were combined, and the Kraft lignin reaction products were isolated using preparative HPLC. This enabled isolation of acetovanillone (4′-hydroxy-3′-methoxyacetophenone, 0.4 mg, 1.0 wt.%), 2,4′-dihydroxy-3′-methoxyacetophenone (1 mg, 2 wt.%), and 3-(4-hydroxy-3-methoxyphenyl)-1-propanol (1.5 mg, 3.0 wt.%). These individual yields are consistent with those determined by GC–MS (Table S3), demonstrating that both the aromatic monomers and CO can be isolated from the reaction. Isolation of the aromatic monomers from lignin is under-addressed in the literature, likely due to the limited number of photocatalytic systems that can cleave lignin into monomers.

### Cleavage of β-O-4 Linkage in Lignin Models

The generation of molecular monomers from lignin was further studied using well-defined molecular models to confirm that they arise from β-O-4 linkage cleavage and determine yields. These experiments were conducted under the same reaction conditions, although the visible-light transparency of the models enabled the use of a monophasic solution that could be contrasted with the bilayer system’s performance. First, focusing on the monophasic conditions, a 2:1 H_2_O/MeCN (3 mL) solvent mixture was used, as the lignin models (5 mM) were insoluble in water. The CdS-AET concentration (1 μM, 0.333 mg mL^–1^) and other parameters were unchanged.

The QDs were first benchmarked against one of the simplest and most-studied model lignin compounds, 2-phenoxy-1-phenylethanol (PP-ol, **1a**, Scheme 1). The CdS-AET enabled >95% conversion of model **1a** to yield phenol **2** (89%) and acetophenone **3** (75%) as the selective β-O-4 cleavage products (Scheme 1 and Table S6). These results match state-of-the-art photocatalytic systems for **1a** cleavage in terms of conversion, yield, and product selectivity (Table S7 contains a literature comparison).^14,19,22,24,39^ Kinetic profiles revealed linear generation of **2** in the first 4 h (0.77 μmol h^–1^, *r* ^2^ = 0.99), with the reaction reaching completion in 16 h (Figure 3). The catalyst turnover number was 475 per QD. Postreaction XPS characterization of the catalyst showed that the Cd 3d_5/2_ and 3d_3/2_, S 2p and O 1s binding energies were unchanged, indicating no apparent photooxidation of the CdS-AET QD surface (Figure S2e).^40^ Exclusion controls confirmed there was no conversion without light or CdS QDs, while control experiments using the as-synthesized oleic acid-capped CdS QDs showed <5% **1a** conversion due to its poor dispersion and instability in the polar solvent mixture (Table S8).

**Scheme 1:**
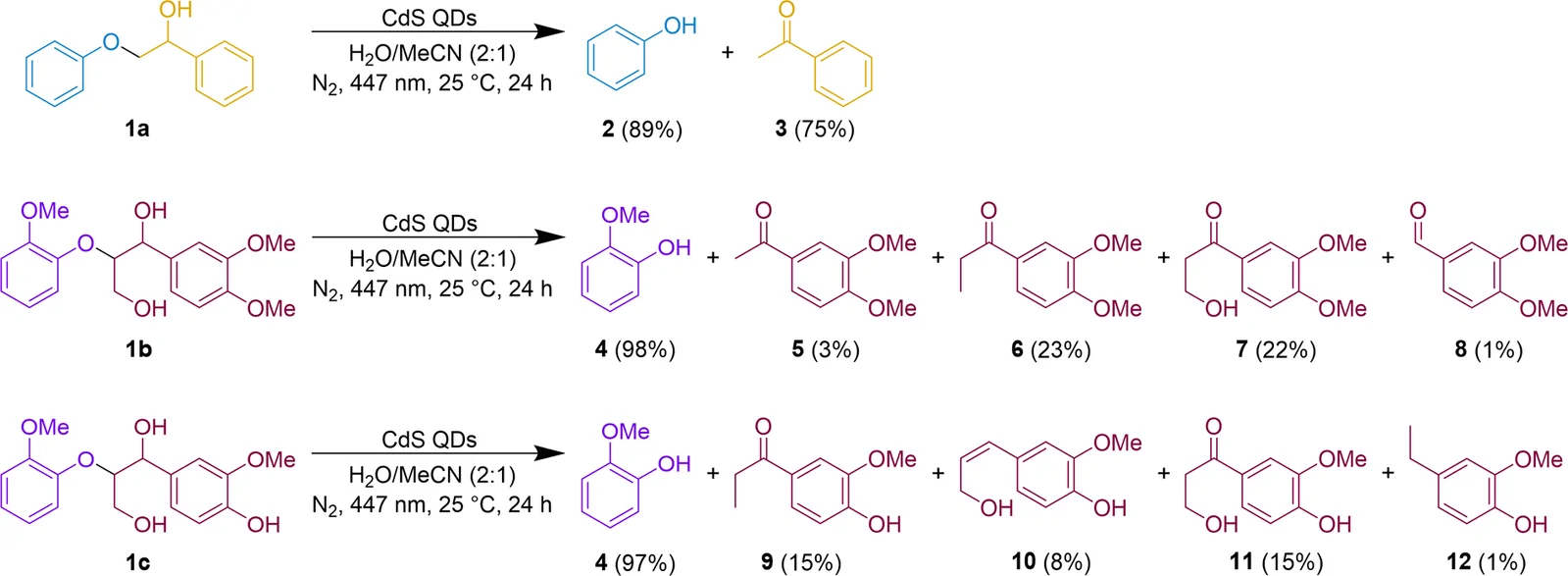
Photocatalytic β-O-4 Cleavage Product Obtained over 24 h under 447 nm, 120 mW cm^–2^, Illumination with CdS-AET QDs (1 μM) in 3 mL of H_2_O/MeCN (2:1, 25 °C, N_2_ Atmosphere, 250 rpm Orbital Shaking) from 5 mM Lignin Model 2-Phenoxy-1-phenylethanol (PP-ol, **1a**), 1-(3,4-dimethoxyphenyl)-2-(2-methoxyphenoxy)-1,3-propanediol (**1b**), and Guaiacyl Glycerol-β-Guaiacyl Ether (**1c**)

**Figure 3:**
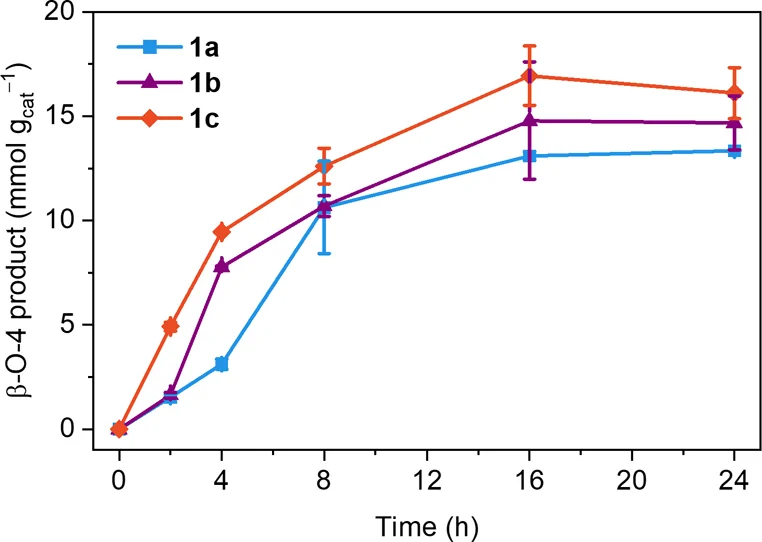
Time profile for the formation of β-O-4 cleavage products from lignin models using CdS-AET QDs. Photocatalytic β-O-4 cleavage product (phenol from 2-phenoxy-1-phenylethanol (PP-ol, **1a**), and guaiacol (**4**) from 1-(3,4-dimethoxyphenyl)-2-(2-methoxyphenoxy)-1,3-propanediol (**1b**) and guaiacyl glycerol-β-guaiacyl ether (**1c**)) (a) (447 nm, 120 mW cm^–2^) over 24 h with CdS-AET QDs (1 μM) in 3 mL H_2_O/MeCN (2:1) from 5 mM lignin models **1a**, **1b**, **1c**. All measurements were performed with 447 nm, 120 mW cm^–2^, illumination at 25 °C under a N_2_ atmosphere with 250 rpm orbital shaking for 24 h, in triplicate, and errors are given as ± one standard deviation.

The model complexity was then increased to be more representative of the lignin β-O-4 linking motifs than **1a** by including additional alkyl chains and hydroxy and methoxy functional groups.^41^ Specifically, this used the “dilignol” type lignin model compounds 1-(3,4-dimethoxyphenyl)-2-(2-methoxyphenoxy)-1,3-propanediol and guaiacyl glycerol-β-guaiacyl ether (**1b** and **1c** in Scheme 1, respectively). Under standard reaction conditions, CdS-AET enabled >95% conversion of each substrate for a quantitative yield of guaiacol (**4**) (98% and 97% for **1b** and **1c**, respectively; Table S6). For both models, the kinetic plots showed linear generation of **4** over the first 4 h (1.72 and 2.38 μmol h^–1^, *r* ^2^ = 0.93 and 0.99, for **1b** and **1c**, respectively), prior to a plateau as the reaction reached completion within 16 h (Figure 3), as was the case for **1a**. This corresponds to 490 turnovers per QD. The other half of the β-O-4 bond resulted in the formation of 3,4-dimethoxy or 3-methoxy,4-hydroxy substituted products from **1b** and **1c**, respectively (**5**–**8** for **1b** and **9**–**12** for **1c**). The aromatic methoxy and hydroxy groups were preserved in all detected products, suggesting that partial degradation of the γ-hydroxyl tail resulted in the formation of multiple products. This observation is consistent with results from the few published systems showing cleavage of “dilignol” compound **1b**, although those studies required elevated temperature and pressure (Table S7).^22,42–44^ This work shows a 65% higher activity per gram of catalyst than the previously published system showing **1b** cleavage at ambient temperature and visible illumination, which also used CdS QDs^20^ (14.7 mmol g_cat_^–1^ of **4** vs 8.9 mmol g_cat_^–1^; Table S7). This difference is attributed to the biphasic light management and full dispersion of the QDs in this work.

In a biphasic configuration with the lignin models in the EtOAc layer (5 mM), as used for Kraft lignin conversion, conversion of the models through β-O-4 cleavage was also observed in all cases (Table S6). A drop in conversion and yield was observed and attributed to the reduced interaction between the photocatalyst and the substrate, which was largely confined to the bilayer interface. In the case of the models, where light competition is not limiting the reaction, having the catalyst and substrate in the same phase expectedly enhances the reaction rate (Table S6).

Examining the lignin model reaction for gaseous products revealed CO generation from the “dilignol” type structure **1b**, but significantly less than from Kraft lignin (0.11 ± 0.03 vs 0.88 ± 0.03 mmol_CO_ g_cat_^–1^; Figure S12). The rate of CO formation did not follow the curve of guaiacol (**4**) formation, indicating two independent reactions. CO generation was lower in **1c**, and only trace amounts were detected in **1a**. No CO was observed in the absence of the model compounds, CdS QDs or light irradiation (Table S9), indicating that the CO is generated from the lignin models via CdS photocatalysis. The distribution of aromatic products was primarily due to variation at the γ-hydroxyl tail, with the lack of selectivity at this site attributed to the varying CO yield from the “dilignol” models.

### Mechanism of CO Evolution from Lignin

Lignin is rich in alcohol and aldehyde groups, which could serve as a source of CO (Figure 1). The different reaction kinetics of CO generation and β-O-4 cleavage during lignin and model conversion also indicated that these were two separate reactions. Therefore, the photocatalytic generation of CO with CdS-AET QDs was probed using alcohol and aldehyde models (2-phenylethanol and 2-phenylacetaldehyde, respectively) in the biphasic system used for lignin conversion.

Photocatalytic tests using 2-phenylethanol and 2-phenylacetaldehyde in place of lignin showed CO generation of 0.26 ± 0.04 and 123 ± 8 mmol g_cat_^–1^ over 24 h, respectively (Figure 4a­(i,ii); Table S10). The higher CO generation by the aldehyde indicates that the predominant CO generation is due to the reaction of CdS-AET with aldehyde motifs in lignin, with a contribution from alcohol groups. This reaction generated toluene in both cases, supporting cleavage of the alpha-beta carbon bond to generate CO. The use of 2-phenylethanoic acid resulted in substantially less CO generation (0.062 ± 0.002 mmol g_cat_^–1^), confirming that the mechanism does not proceed via aldehyde oxidation to a carboxylic acid followed by decarboxylation and CO_2_ reduction to CO. This is consistent with only trace CO_2_ (<0.5% of CO) being detected, while the absence of formate indicates a mechanism in which it is not an intermediate either. Tests with anisole yielded negligible CO, indicating that the methoxy groups in lignin are not contributing to CO (Table S10). Furthermore, substituting the formyl proton on the aldehyde with a methyl group by replacing phenylacetaldehyde with phenylacetone also suppresses the reaction 400-fold (123 ± 8 vs 0.30 ± 0.02 mmol g_cat_^–1^; Figure 4a­(iii), and Table S10), supporting that the formyl hydrogen is involved in the reaction.

**Figure 4:**
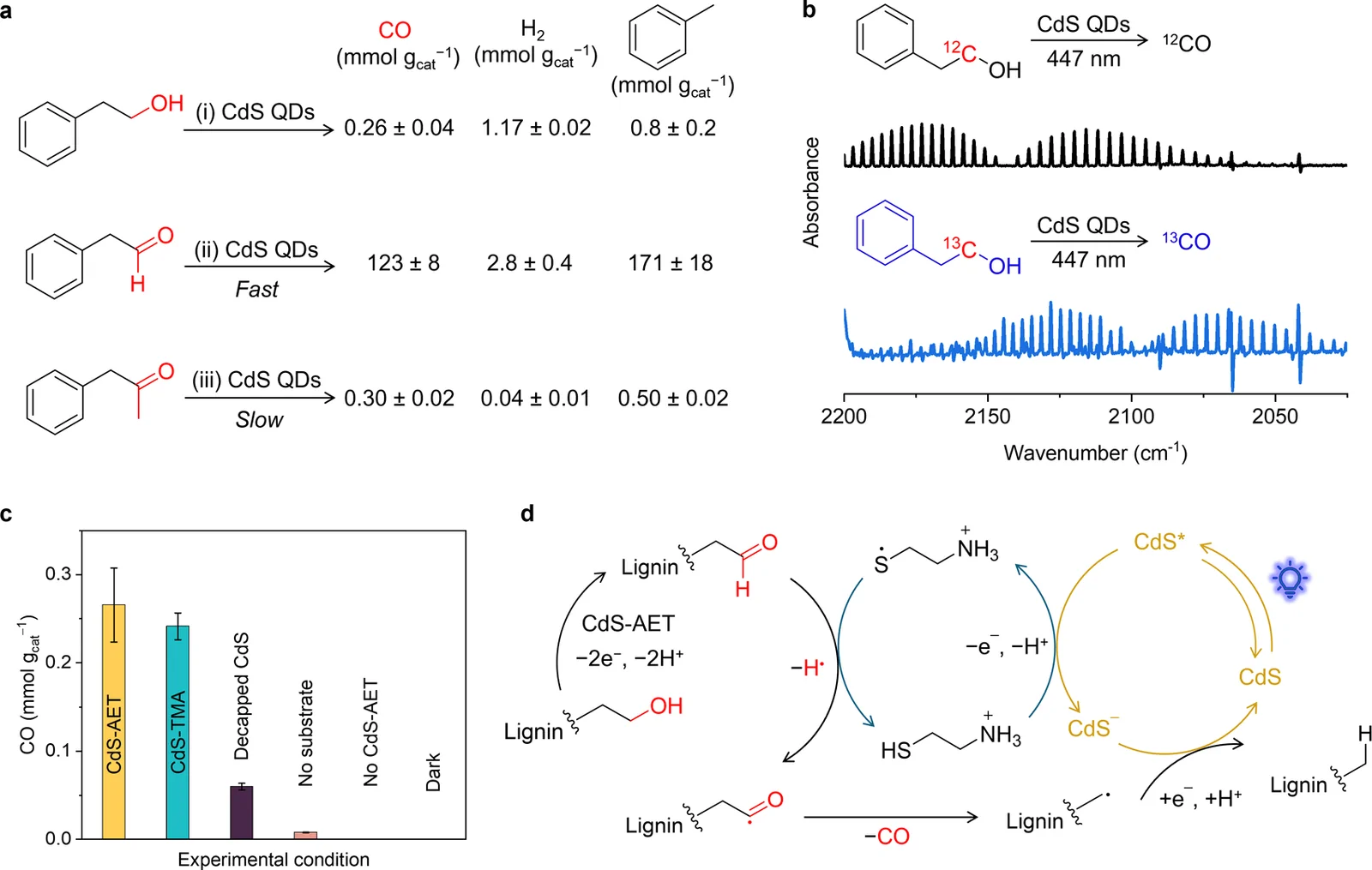
Mechanistic study for lignin photoreforming to CO using CdS-AET QDs. (a) Control experiments with 0.5 M 2-phenylethanol, 2-phenylacetaldehyde and phenylacetone using CdS-AET QDs (1 μM) in 3 mL H_2_O/EtOAc (1:1). (b) Transmission IR spectra of the headspace gas obtained after photocatalysis with 0.5 M 2-phenyl­[1-^12^C]­ethanol (black) and 2-phenyl­[1-^13^C]­ethanol (blue) over 24 h. (c) CO production using 1 μM CdS QDs under different capping conditions with 0.5 M 2-phenylethanol along with exclusion controls in 3 mL H_2_O/EtOAc (1:1). All measurements were performed with 447 nm, 120 mW cm^–2^, illumination at 25 °C under a N_2_ atmosphere with 250 rpm orbital shaking for 24 h, in triplicate, and errors are given as ± one standard deviation. (d) Proposed mechanism for photocatalytic lignin reforming to CO using CdS-AET QDs.

The CdS-AET catalyst can oxidize alcohols to aldehydes under photocatalytic conditions;^45^ thus, CO generation via alcohols and aldehydes was probed with an isotopic-labeling experiment using 2-phenyl­[1-^13^C]­ethanol. This reaction generated ^13^CO in the headspace, which was detected by gas-phase transmission infrared spectroscopy (Figure 4b), thereby confirming that alcohol moieties in lignin, alongside aldehyde motifs, could be converted to CO.

The formation of CO from 2-phenylethanol was suppressed in the presence of either electron or hole scavengers (K_2_S_2_O_8_ or Na_2_SO_3_, respectively), indicating the participation of both photogenerated holes and electrons in the reaction. The addition of the radical trap 5,5-dimethyl-1-pyrroline-*N*-oxide (DMPO) enabled identification of the 2-phenylethanol and 2-phenylacetaldehyde adducts by ESI-MS (Table S10), supporting a radical mechanism.

The importance of the QD capping ligands was then studied, given that thiol groups are known to catalyze hydrogen atom transfer (HAT) reactions,^46^ while also ruling out any potential contribution from the (protonated) amine. The influence of both the thiol and the protonated amine was probed by excluding AET and not using a QD capping ligand. Although the decapped CdS QDs resulted in similar β-O-4 cleavage from the lignin model compound (**1a**, PP-ol) and Kraft lignin as CdS-AET (>95% PP-ol conversion for both CdS-AET and decapped CdS; Table S8), the CO generation decreased 4-fold from Kraft lignin, confirming that the AET ligand is not active for β-O-4 cleavage but is fundamental for CO generation (Figure 4c). These results further support the occurrence of two parallel, independent processes in the system. Sulfur cannot be entirely excluded due to its presence in the CdS structure, and the residual CO generation absent AET may be due to reaction of surface sulfur in an analogous manner. The influence of the amine was excluded by exchanging the AET ligand with trimethylated amine (2-mercaptoethyl-*N*,*N*,*N*-trimethylammonium chloride; TMA), which revealed no change compared to the standard AET capping. Further exclusion controls confirmed that CO evolution required the presence of all the components, including CdS-AET, light and one of 2-phenylethanol or 2-phenylacetaldehyde (Figure 4c). Thus, the thiol group in the capping ligand was identified as key to CO generation from the alcohol groups.

The catalytic nature of the AET capping ligand was confirmed via recyclability tests. While monomer generation was unaffected in the 10-cycle stability test, CO generation dropped after the first cycle and did not recover upon the addition of a fresh Kraft lignin solution. However, adding catalytic amounts of AET (60 μM; 0.09 μmol) to the recycled CdS QDs after each run recovered the CO generation for subsequent photocatalysis cycles. This addition confirms that AET is catalytic with an initial turnover number of 5 (Figure S13), which is within the typical range for a HAT catalyst (where 10–20 mol % is common). It is likely that the TON is limited by irreversible oxidation or radical coupling processes.^45^ The differing TON for β-O-4 cleavage and CO generation, alongside the differing rates, indicates these reactions occur in parallel.

Combining these results with literature precedent, a decarbonylation mechanism is proposed for CO generation from lignin. For alcohol groups, the first step is the two-electron oxidation of the alcohol to an aldehyde by CdS (Figure 4d). A separate oxidation of a thiol ligand by CdS generates a thiyl radical that can act as a HAT catalyst by abstracting the formyl hydrogen from the aldehyde, which regenerates the thiol and completes the HAT catalysis cycle. Spontaneous homolytic cleavage of the acyl radical then releases the observed CO. The residual radical is then reduced to close the cycle.

This mechanistic cycle is consistent with the observations, first with alcohol oxidation to an aldehyde being well-precedented for CdS, the ^13^C labeled alcohol generating ^13^CO, and the reaction rate accelerating when aldehyde was added directly. The thiol HAT cycle is consistent with the importance of the AET ligand to CO generation, and CdS is known to oxidize thiols. It is likely that HAT from the carbon alpha (or beta with rearrangement) to the alcohol group occurs competitively with direct alcohol oxidation by CdS, with this mediated pathway also contributing to the observed activity. The transfer of the formyl hydrogen of an aldehyde to a thiyl radical is established,^47,48^ as is the decarbonylation of an acyl radical to release CO.^47^ The generation of toluene and trapping of the toluyl radical intermediate also support the mechanism. Rate suppression by addition of oxidative and reductive scavengers supports the mechanism having reductive and oxidative steps that affect the rate. The final balancing of charges via H_2_ evolution is observed for the model compounds but not for lignin (no H_2_ detected; Figure 4a), with the electron balancing attributed to other, undefined reductions within lignin subunits during the cleavage reaction. The nature of these processes cannot be identified due to the complexity and heterogeneity of units in polymeric lignin, but the measurement of a H_2_-free CO stream confirms that the overall reaction is charge-balanced.

## Conclusions

We have introduced a biphasic system that interfaces a photocatalyst with lignin without direct light competition. This system uses aminoethanethiol-capped CdS QDs for the photocatalytic conversion of lignin into both CO and functionalized aromatics under visible light at ambient temperatures and pressures. The CO was generated from Kraft lignin as well as raw biomass samples as a H_2_-free gas stream. We have demonstrated the utilization of CO for carbonylative cross-coupling in a compartmentalized cascade reaction. Therein, CO was generated from Kraft lignin and utilized for aminocarbonylation in one pot under visible light.

Concurrently, the aromatic monomers were obtained from β-O-4 bond cleavage of Kraft lignin with 81% yield (19 wt % of starting lignin). Equivalent yields of CO and monomers were obtained under LED illumination and simulated solar light. The high aromatic yields were supported by >95% conversion of defined model compounds containing β-O-4 linkers. A mechanistic study identified hydroxyl and aldehyde groups in lignin as the primary source of CO. This reaction proceeds via alcohol oxidation to the corresponding aldehyde, followed by hydrogen atom transfer catalyzed by the thiol ligand to generate an acyl radical, which cleaves to yield CO in a decarbonylation step. These results establish a proof of concept for generating a green CO feedstock from lignin under mild reaction conditions, alongside aromatic monomers, with state-of-the-art yields, and utilizing this biomass-derived CO for organic synthesis.

## Experimental Section

### Chemicals

The syntheses of 2-mercaptoethyl-*N*,*N*,*N*-trimethylammonium chloride (TMA),^49^ decapped CdS QDs,^50^ 1-phenylpropan-2-one,^51^ 2-phenyl­[1-^13^C]­ethanol,^52–54^ and 4-methoxy-*N*-octylbenzamide^38^ were adapted from literature procedures with characterization in the SI. Commercial chemicals were used as supplied with details in the Supporting Information.

### Synthesis of CdS Quantum Dots

Oleic-acid-capped CdS QDs (CdS-OA) were synthesized using an established hot-injection procedure with slight modifications.^33,55^ A flask containing CdO (0.64 g), oleic acid (29 mL), and octadecene (89 mL), and one containing sulfur (0.08 g) in octadecene (20 mL), were each degassed under vacuum (50 °C, 1 h) and placed under N_2_. The Cd-containing solution was heated to 280 °C, and half of the sulfur solution was rapidly injected therein, while the other half was added over 2 min, followed by cooling to 220 °C under N_2_ flow and quenching. The cooled mixture was diluted with 1:1 hexane/methanol (100 mL) before precipitation with acetone (300 mL), isolation by centrifugation (10k rpm, 10 min), and redispersal in hexane (precipitation and isolation repeated twice). The concentration of the QDs was determined by UV–vis absorption spectroscopy using the average particle size and the extinction coefficient calculated from the first absorption peak at 462 nm.^56^

### Ligand Exchange of CdS Quantum Dots

2-Aminoethanethiol·HCl in H_2_O (5 M, 5 mL) was added to CdS-OA in hexane (1 mL) at rt and stirred (1 h). Additional ligand (5 M in H_2_O, ∼ 3 mL) was then added until a phase transfer of the particles from hexane to H_2_O was achieved, after which the QDs were isolated by centrifugation (10k rpm, 10 min), washed with acetone, and redispersed in H_2_O (Figure S3). The same procedure was used with 2-mercaptoethyl-*N*,*N*,*N*-trimethylammonium chloride (TMA).

### Photocatalytic Experiments

For biphasic testing, solutions were prepared containing CdS QDs in H_2_O (1 μM, 1.5 mL) with a layer of EtOAc containing the lignin model compound (5 mM), Kraft lignin, or a raw lignin source (1.5 mL) in a crimp-cap vial (11.7 mL volume). Monophasic solutions were prepared by dispersing CdS QDs (1 μM, diluted from stock solution) and the substrate (5 mM) in a 2:1 v/v mixture of H_2_O and MeCN (3 mL). The crimp-cap vial was sealed with a septum and purged with N_2_ (containing 2% CH_4_ as an internal standard for GC analysis) for 10 min. Vials were then placed in an LED photoreactor (Treellum Technologies) and illuminated with a blue LED (447 nm, 120 mW cm^–2^) with orbital convection (250 rpm) at 25 °C and 1 bar pressure for 24 h. Tests under simulated solar light (air mass 1.5 global filter, AM 1.5G) were done using a solar light simulator (Newport Oriel, 100 mW cm^–2^).

Biphasic carbonylation cascade reactions were performed using the same biphasic testing solutions as for CO generation from Kraft lignin (CdS QDs in H_2_O (1 μM, 1.5 mL) with EtOAc containing Kraft lignin (1.5 mL) in a crimp-cap vial (11.7 mL volume, headspace 8.7 mL)), but with additional carbonylation reagents (varied under optimization conditions, Table S2) in the EtOAc layer. Carbonylation reaction in a two-compartment reactor (7.5 mL volume, headspace 2.5 mL) was performed with one compartment containing the biphasic solution for CO generation (CdS QDs in H_2_O (1 μM, 1.5 mL) with EtOAc containing Kraft lignin (1.5 mL)) and the other compartment containing carbonylation reagents in EtOAc (2 mL) (Figure S9).

### Product Analysis

Gaseous products (H_2_ and CO) were quantified through headspace gas analysis (100 μL) using a Shimadzu GC-2010 Plus gas chromatograph equipped with an ionization detector and molecular sieve column. Methane (2% in N_2_) was used as an internal standard for all the gas analysis experiments, and the corresponding response factors were determined through calibration using known amounts of H_2_, CO, and CH_4_.

Conversion of model lignin compounds was quantified with high-performance liquid chromatography (Waters Breeze with 210 nm UV detector) using isocratic flow (1 mL min^–1^) through a C18 column at 40 °C; specifically, 2-phenoxy-1-phenylethanol (**1a**; using 3:2 H_2_O/CH_3_CN), 1-(3,4-dimethoxyphenyl)-2-(2-methoxyphenoxy)-1,3-propanediol (**1b**; 3:2 H_2_O/MeOH), and guaiacyl glycerol-β-guaiacyl ether (**1c**; 3:2 H_2_O/MeOH), each using calibration curves determined using external standards (Figure S14). CdS QDs were aggregated (acetone) and separated (centrifugation at 10k rpm, 10 min) before chromatography. Details of preparative HPLC in Supporting Information.

Product yields from model lignin compounds, Kraft lignin, and carbonylation were analyzed by GC–MS (Agilent 5977C with TCD, FID and EI-MS detectors and a HP-5MS capillary column). The FID detector and the effective carbon number (ECN) method (Table S3), with the addition of an internal standard solution of *n*-decane (1 mM), were used for quantification, and MS was used for identification. EI-MS was used for the quantification of the aminocarbonylation product. Temperature program: injection at 250 °C, column at 80 °C (2 min), ramp to 200 °C (20 °C min^–1^), hold (3 min), ramp to 260 °C (20 °C min^–1^) and hold (11 min).

## Supplementary Material



## Data Availability

Primary data supporting the findings of this study are available from the University of Cambridge open-access data repository (https://doi.org/10.17863/CAM.133291) with additional information available from the corresponding authors upon reasonable request.

## Notes

Elaboration on the characterization of CdS quantum dots; additional experimental details and characterization; tabulated values from photocatalytic testing, carbonylation cascades, lignin and model monomer quantification, NMR assignment, literature comparison and control experiments; CdS characterization by UV–vis, TEM, XPS, GCMS results from the carbonylation cascade and lignin monomers, lignin 2D NMR, CO generation results from model complexes, and HPLC calibration curves (PDF)

## References

[1] Ragauskas A. J., Beckham G. T., Biddy M. J., Chandra R., Chen F., Davis M. F., Davison B. H., Dixon R. A., Gilna P., Keller M., Langan P., Naskar A. K., Saddler J. N., Tschaplinski T. J., Tuskan G. A., Wyman C. E. (2014). Lignin Valorization:
Improving Lignin Processing in the Biorefinery. Science.

[2] Tuck C. O., Pérez E., Horváth I. T., Sheldon R. A., Poliakoff M. (2012). Valorization
of Biomass: Deriving More Value from Waste. Science.

[3] Rinaldi R., Jastrzebski R., Clough M. T., Ralph J., Kennema M., Bruijnincx P. C. A., Weckhuysen B. M. (2016). Paving the Way for Lignin Valorisation:
Recent Advances in Bioengineering, Biorefining and Catalysis. Angew. Chem., Int. Ed..

[4] Zinoviev S., Müller-Langer F., Das P., Bertero N., Fornasiero P., Kaltschmitt M., Centi G., Miertus S. (2010). Next-Generation Biofuels:
Survey of Emerging Technologies and Sustainability Issues. ChemSusChem.

[5] Okolie J. A., Mukherjee A., Nanda S., Dalai A. K., Kozinski J. A. (2021). Next-Generation
Biofuels and Platform Biochemicals from Lignocellulosic Biomass. Int. J. Energy Res..

[6] Liu M., Han B., Dyson P. J. (2022). Simultaneous Generation of Methyl Esters and CO in
Lignin Transformation. Angew. Chem., Int. Ed..

[7] Li Z., Sutandar E., Goihl T., Zhang X., Pan X. (2020). Cleavage of
Ethers and Demethylation of Lignin in Acidic Concentrated Lithium
Bromide (ACLB) Solution. Green Chem..

[8] Sturgeon M. R., Kim S., Lawrence K., Paton R. S., Chmely S. C., Nimlos M., Foust T. D., Beckham G. T. (2014). A Mechanistic Investigation of Acid-Catalyzed
Cleavage of Aryl-Ether Linkages: Implications for Lignin Depolymerization
in Acidic Environments. ACS Sustainable Chem.
Eng..

[9] Brienza F., Cannella D., Montesdeoca D., Cybulska I., Debecker D. P. (2024). A Guide
to Lignin Valorization in Biorefineries: Traditional, Recent, and
Forthcoming Approaches to Convert Raw Lignocellulose into Valuable
Materials and Chemicals. RSC Sustainability.

[10] Sun Z., Bottari G., Afanasenko A., Stuart M. C. A., Deuss P. J., Fridrich B., Barta K. (2018). Complete Lignocellulose
Conversion
with Integrated Catalyst Recycling Yielding Valuable Aromatics and
Fuels. Nat. Catal..

[11] Shuai L., Amiri M. T., Questell-Santiago Y.
M., Héroguel F., Li Y., Kim H., Meilan R., Chapple C., Ralph J., Luterbacher J. S. (2016). Formaldehyde Stabilization Facilitates Lignin Monomer
Production during Biomass Depolymerization. Science.

[12] Zakzeski J., Bruijnincx P. C. A., Jongerius A. L., Weckhuysen B. M. (2010). The Catalytic
Valorization of Lignin for the Production of Renewable Chemicals. Chem. Rev..

[13] Kasap H., Achilleos D. S., Huang A., Reisner E. (2018). Photoreforming of Lignocellulose
into H_2_ Using Nanoengineered Carbon Nitride under Benign
Conditions. J. Am. Chem. Soc..

[14] Liu H., Li H., Lu J., Zeng S., Wang M., Luo N., Xu S., Wang F. (2018). Photocatalytic Cleavage of C–C Bond in Lignin
Models under Visible Light on Mesoporous Graphitic Carbon Nitride
through π–π Stacking Interaction. ACS Catal..

[15] Ku C., Li K., Guo H., Wu Q., Yan L. (2022). One-Step Construction
of Mesoporous Cyano and Sulfur Co-Modified Carbon Nitride for Photocatalytic
Valorization of Lignin to Functionalized Aromatics. Appl. Surf. Sci..

[16] Wakerley D. W., Kuehnel M. F., Orchard K. L., Ly K. H., Rosser T. E., Reisner E. (2017). Solar-Driven Reforming
of Lignocellulose to H_2_ with a CdS/CdO_x_ Photocatalyst. Nat. Energy.

[17] Guo Y., Chen X., Liu Y., Chen Z., Guo P., Luo D., Zhang M., Liu X. (2024). Inorganic–Organic Dual-Ligand-Regulated
Photocatalysis of CdS@Zn_x_Cd_1–x_S@ZnS Quantum
Dots for Lignin Valorization. ACS Appl. Mater.
Interfaces.

[18] Wu X., Xie S., Liu C., Zhou C., Lin J., Kang J., Zhang Q., Wang Z., Wang Y. (2019). Ligand-Controlled Photocatalysis
of CdS Quantum Dots for Lignin Valorization under Visible Light. ACS Catal..

[19] Han G., Yan T., Zhang W., Zhang Y. C., Lee D. Y., Cao Z., Sun Y. (2019). Highly Selective Photocatalytic Valorization of Lignin
Model Compounds
Using Ultrathin Metal/CdS. ACS Catal..

[20] Wu X., Fan X., Xie S., Lin J., Cheng J., Zhang Q., Chen L., Wang Y. (2018). Solar Energy-Driven
Lignin-First
Approach to Full Utilization of Lignocellulosic Biomass under Mild
Conditions. Nat. Catal..

[21] Tang J.-P., Chen Y., Wang Z.-Y., Hu Y.-H., Wang J.-H., Bao L., Zhao Z.-Y., Yuan Y.-J. (2025). Sustainable
H_2_ Production
from Lignocellulosic Biomass over MoS_2_ Modified Sulfur
Vacancy Enriched ZnIn_2_S_4_ Photocatalyst. ACS Catal..

[22] Chen L., Liu Y., Mitra S., Kim D., Huang Z., Vahey D. M., Bin Mohamad Annuar A., Reisner E. (2025). Solar Lignin Reforming with Tunable
Selectivity Using a Hybrid Photocatalyst in Aqueous Solution. J. Am. Chem. Soc..

[23] Luo N., Wang M., Li H., Zhang J., Liu H., Wang F. (2016). Photocatalytic Oxidation–Hydrogenolysis of Lignin β-O-4
Models via a Dual Light Wavelength Switching Strategy. ACS Catal..

[24] Luo N., Wang M., Li H., Zhang J., Hou T., Chen H., Zhang X., Lu J., Wang F. (2017). Visible-Light-Driven
Self-Hydrogen Transfer Hydrogenolysis of Lignin Models and Extracts
into Phenolic Products. ACS Catal..

[25] Lam E., Reisner E. (2021). A TiO_2_-Co­(Terpyridine)_2_ Photocatalyst
for the Selective Oxidation of Cellulose to Formate Coupled to the
Reduction of CO_2_ to Syngas. Angew.
Chem., Int. Ed..

[26] Speltini A., Sturini M., Dondi D., Annovazzi E., Maraschi F., Caratto V., Profumo A., Buttafava A. (2014). Sunlight-Promoted
Photocatalytic Hydrogen Gas Evolution from Water-Suspended Cellulose:
A Systematic Study. Photochem. Photobiol. Sci..

[27] Srisasiwimon N., Chuangchote S., Laosiripojana N., Sagawa T. (2018). TiO_2_/Lignin-Based
Carbon Composited Photocatalysts for Enhanced Photocatalytic Conversion
of Lignin to High Value Chemicals. ACS Sustainable
Chem. Eng..

[28] Yuan L., Qi M.-Y., Tang Z.-R., Xu Y.-J. (2021). Coupling Strategy
for CO_2_ Valorization Integrated with Organic Synthesis
by Heterogeneous Photocatalysis. Angew. Chem.,
Int. Ed..

[29] Jing Y.-N., Wang H.-X., Wang C., Ye C., Tung C.-H., Wu L.-Z. (2025). A Highly Efficient Molecular Iron­(II) Photocatalyst for Concurrent
CO_2_ Reduction and Organic Synthesis. J. Am. Chem. Soc..

[30] Jensen M. T., Rønne M. H., Ravn A. K., Juhl R. W., Nielsen D. U., Hu X.-M., Pedersen S. U., Daasbjerg K., Skrydstrup T. (2017). Scalable Carbon Dioxide Electroreduction Coupled to
Carbonylation Chemistry. Nat. Commun..

[31] Xu Y., Schoonen M. A. A. (2000). The Absolute
Energy Positions of Conduction and Valence
Bands of Selected Semiconducting Minerals. Am.
Mineral..

[32] Zhao J., Holmes M. A., Osterloh F. E. (2013). Quantum
Confinement Controls Photocatalysis:
A Free Energy Analysis for Photocatalytic Proton Reduction at CdSe
Nanocrystals. ACS Nano.

[33] Huang L., Wang X., Yang J., Liu G., Han J., Li C. (2013). Dual Cocatalysts Loaded Type I CdS/ZnS Core/Shell Nanocrystals as
Effective and Stable Photocatalysts for H_2_ Evolution. J. Phys. Chem. C.

[34] Boles M. A., Ling D., Hyeon T., Talapin D. V. (2016). The Surface Science
of Nanocrystals. Nat. Mater..

[35] Byrne F. P., Jin S., Paggiola G., Petchey T. H. M., Clark J. H., Farmer T. J., Hunt A. J., Robert McElroy C., Sherwood J. (2016). Tools and Techniques
for Solvent Selection: Green Solvent Selection Guides. Sustain Chem. Process.

[36] Xia Y.-S., Tang M., Zhang L., Liu J., Jiang C., Gao G.-K., Dong L.-Z., Xie L.-G., Lan Y.-Q. (2022). Tandem
Utilization of CO_2_ Photoreduction Products for the Carbonylation
of Aryl Iodides. Nat. Commun..

[37] Mitra S., Vahey D. M., Bonke S. A., Reisner E. (2025). Compartmentalised
Carbonylation
Cascade Initiated by Photocatalytic CO_2_ Reduction. ChemRxiv.

[38] Martinelli J. R., Watson D. A., Freckmann D. M. M., Barder T. E., Buchwald S. L. (2008). Palladium-Catalyzed
Carbonylation Reactions of Aryl Bromides at Atmospheric Pressure:
A General System Based on Xantphos. J. Org.
Chem..

[39] Xu X., Dai S., Xu S., Zhu Q., Li Y. (2023). Efficient Photocatalytic
Cleavage of Lignin Models by a Soluble Perylene Diimide/Carbon Nitride
S-Scheme Heterojunction. Angew. Chem., Int.
Ed..

[40] Pechstedt K., Whittle T., Baumberg J., Melvin T. (2010). Photoluminescence of
Colloidal CdSe/ZnS Quantum Dots: The Critical Effect of Water Molecules. J. Phys. Chem. C.

[41] Lahive C. W., Kamer P. C. J., Lancefield C. S., Deuss P. J. (2020). An Introduction
to Model Compounds of Lignin Linking Motifs; Synthesis and Selection
Considerations for Reactivity Studies. ChemSusChem.

[42] Wang M., Lu J., Li L., Li H., Liu H., Wang F. (2017). Oxidative
C­(OH)C Bond Cleavage of Secondary Alcohols to Acids over a Copper
Catalyst with Molecular Oxygen as the Oxidant. J. Catal..

[43] Wang M., Lu J., Zhang X., Li L., Li H., Luo N., Wang F. (2016). Two-Step, Catalytic
C–C Bond Oxidative Cleavage Process Converts
Lignin Models and Extracts to Aromatic Acids. ACS Catal..

[44] Rinesch T., Mottweiler J., Puche M., Concepción P., Corma A., Bolm C. (2017). Mechanistic Investigation of the
Catalyzed Cleavage for the Lignin β-O-4 Linkage: Implications
for Vanillin and Vanillic Acid Formation. ACS
Sustainable Chem. Eng..

[45] Zhao L.-M., Meng Q.-Y., Fan X.-B., Ye C., Li X.-B., Chen B., Ramamurthy V., Tung C.-H., Wu L.-Z. (2017). Photocatalysis
with Quantum Dots and Visible Light: Selective and Efficient Oxidation
of Alcohols to Carbonyl Compounds through a Radical Relay Process
in Water. Angew. Chem., Int. Ed..

[46] Bao X., Yu W., Wang G. (2023). Thiols as
Powerful Atom Transfer Catalyst: Opportunities
in Photoredox-Mediated Reactions. Adv. Synth.
Catal..

[47] Chatgilialoglu C., Crich D., Komatsu M., Ryu I. (1999). Chemistry of Acyl Radicals. Chem. Rev..

[48] Yoshikai K., Hayama T., Nishimura K., Yamada K., Tomioka K. (2005). Thiol-Catalyzed
Acyl Radical Cyclization of Alkenals. J. Org.
Chem..

[49] Chalker J. M., Lercher L., Rose N. R., Schofield C. J., Davis B. G. (2012). Conversion of Cysteine into Dehydroalanine
Enables
Access to Synthetic Histones Bearing Diverse Post-Translational Modifications. Angew. Chem., Int. Ed..

[50] Chang C. M., Orchard K. L., Martindale B. C. M., Reisner E. (2016). Ligand Removal from
CdS Quantum Dots for Enhanced Photocatalytic H_2_ Generation
in pH Neutral Water. J. Mater. Chem. A.

[51] Roure B., Alonso M., Lonardi G., Yildiz D. B., Buettner C. S., Dos Santos T., Xu Y., Bossart M., Derdau V., Méndez M., Llaveria J., Ruffoni A., Leonori D. (2025). Photochemical
Permutation of Thiazoles, Isothiazoles and Other Azoles. Nature.

[52] Gilman H., Catlin W. E. (1925). n-PROPYLBENZENE. Org. Synth..

[53] Lee C. C., Zohdi H. F., Sallam M. M. M. (1985). Hydrogen-Deuterium
Exchanges in a
Friedel-Crafts Reaction. J. Org. Chem..

[54] Nakamura K., Nakajima T., Aoyama T., Okitsu S., Koyama M. (2014). One-Pot Esterification
and Amidation of Phenolic Acids. Tetrahedron.

[55] Murray C. B., Norris D. J., Bawendi M. G. (1993). Synthesis
and Characterization of
Nearly Monodisperse CdE (E = Sulfur, Selenium, Tellurium) Semiconductor
Nanocrystallites. J. Am. Chem. Soc..

[56] Yu W. W., Qu L., Guo W., Peng X. (2003). Experimental Determination of the
Extinction Coefficient of CdTe, CdSe, and CdS Nanocrystals. Chem. Mater..

