# Supplementary material for: Biphasic Solar Reforming of Lignin to Aromatics and CO for In Situ Carbonylation of Organics
Source: J Am Chem Soc. 2026 Aug 28;148(35):38153–62. doi: 10.1021/jacs.6c10525 (PMC13564410; doi:10.1021/jacs.6c10525)
Supplement: Supplementary file 1 [file ja6c10525_si_001.pdf]

Supporting Information for

Biphasic Solar Reforming of Lignin to Aromatics and CO for in situ Carbonylation of Organics

Diksha Mittal, Sampurna Mitra, Etienne Bonvin, David R. Spring, Shannon A. Bonke,* Erwin Reisner*

Yusuf Hamied Department of Chemistry, University of Cambridge, Lensfield Rd, Cambridge CB2 1EW, UK.

* Correspondence to: Dr S. A. Bonke (sabonke@ch.cam.ac.uk) and Prof E. Reisner (reisner@ch.cam.ac.uk).

Contents

Supporting Note 1: Characterization of Aqueous CdS Quantum Dots	S2
Additional Experimental Section	S3
Supporting Tables	S9
Supporting Figures	S21
References	S30

Supplementary Note 1: Characterization of Aqueous CdS Quantum Dots

CdS QDs were first synthesized using the hot-injection method and dispersed in hexane with oleic acid ligands (CdS-OA).[1] The tunability of the organic ligand was further explored to enable the dispersion of the QDs in desirable solvent systems.[2] A ligand exchange procedure was then used to transfer the QDs into aqueous media that can stabilize the charges generated during redox catalysis. This exchange of the hydrophobic oleic acid ligand with hydrophilic 2-aminoethanethiol (AET) ligand transferred the CdS QDs from apolar organic to aqueous media (CdS-AET; **Figure S2a** and **S3**).

UV-vis absorption spectroscopy showed that both the as-synthesized CdS-OA in hexane and CdS-AET in water showed a characteristic first excitonic peak at 462 nm (**Figure S2b**), indicating a particle size of ~5.8 nm.[3] Negligible scattering was observed at higher wavelengths to indicate that there was no size alteration or aggregation during the ligand exchange. Transmission electron micrographs confirmed the QDs had the expected size with no difference between the CdS-OA and CdS-AET particles (5.9 ± 0.9 vs 5.6 ± 0.7 nm; **Figure S2c,d** and **S4**).

X-ray photoelectron spectroscopy (XPS) revealed binding energies consistent with literature values for CdS QDs and no differences in the CdS-OA and CdS-AET sample binding energies in the Cd $3d_{5/2}$ and $3d_{3/2}$ (405 and 412 eV), S 2p (161.6 eV) and O 1s (532.7 eV) regions (**Figure S2e**, and **S5** for O 1s and S 2p spectra).[4] This indicated no oxidation of the CdS-AET QD surface upon ligand exchange and confirmed the structural integrity and stability of the QDs in polar media.

Additional Experimental Methods

Reagents.

CdO (99%, Thermo Scientific), sulfur (99.99%, Thermo Scientific), $NH_2(CH_2)_2SH$•HCl (99%, Fluorochem), 2-phenoxy-1-phenylethanol (96%, Fluorochem), 1-(3,4-dimethoxyphenyl)-2-(2-methoxyphenoxy)propane-1,3-diol (95%, Fluorochem), guaiacylglycerol-beta-guaiacyl ether (>97.0%, Tokyo Chemical Industry), Kraft lignin (Sigma-Aldrich), 2-phenylethanol (99%, Sigma-Aldrich), 2-phenylacetaldehyde (95%, Fluorochem), phenylacetic acid (98.5%, Thermo Scientific), 3,4-dihydro-2,3-dimethyl-2H-pyrrole 1-oxide (98%, Cayman Chemical Company), benzyl chloride (99%, Sigma-Aldrich), magnesium (99.5%, Sigma-Aldrich), and $LiAlH_4$ (1 M in THF, Thermo Scientific) were obtained commercially and used without further purification. The assorted lignin samples were sourced from Cambridge, UK and ground before use. The reaction gas (N_2 with 2% CH_4) was purchased from BOC, and $^{13}CO_2$ was acquired from Sigma-Aldrich.

Physical Characterization.

UV-Vis absorption spectra were recorded on an Agilent Cary 60 UV-Vis spectrophotometer. ^{1}H- and ^{13}C-NMR spectra were collected on a Bruker 700 MHz Avance spectrometer at room temperature, unless specified otherwise. FT-IR spectroscopy was performed on a PerkinElmer Spectrum 3 FT-IR spectrometer. TEM used a Thermo Scientific Talos F200X G2 operated at 200kV using a Thermo Scientific Ceta CMOS camera. XPS used a Thermo Scientific Escalab 250Xi equipped with a monochromated Al kα X-ray source (1486.7 eV). The data were acquired at room temperature (298 K) and at a pressure below 10^{-8} Torr. Mass Spectrometric analyses (using Waters Xevo G2-S, Waters Synapt G2-Si) were conducted by the Mass Spectrometry Services of the Yusuf Hamied Department of Chemistry, University of Cambridge.

Synthesis of decapped (ligand-free) CdS quantum dots (CdS-BF_4).

The synthesis was adapted from a reported literature procedure.[5] Oleic acid-capped CdS quantum dots (CdS-OA QDs) in hexane (1 mL) had solvent removed in vacuo and were redispersed in a mixture of anhydrous chloroform (15 mL) and anhydrous DMF (2 mL) under N_2. Et_3OBF_4 (1.0 M in DCM, 9 mL) was added, and the reaction mixture was stirred at rt (1 h). Me_3OBF_4 (1.0 M in MeCN) was then added gradually until the particles precipitated (~5 mL), at which point the QDs were isolated by centrifugation (8k rpm,

7 min) and redispersed in DMF (**Figure S15**). The photocatalytic tests with CdS-BF_4 were carried out in aqueous dispersion by first removing DMF under reduced pressure.

Synthesis of 2-mercaptoethyl-N,N,N-trimethylammonium chloride (TMA) ligand.

The synthesis was adapted from the reported procedure.[6]

Synthesis of 2-(acetothioethyl)-trimethylammonium bromide

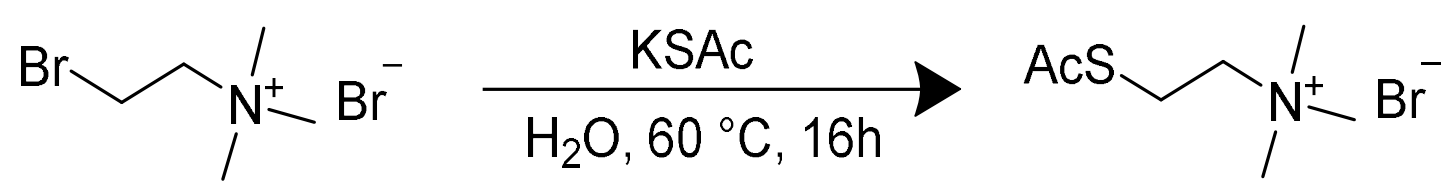

Potassium thioacetate (3.0 g, 26.3 mmol) was added to 2-(bromoethyl)-trimethylammonium bromide (5.0 g, 20.2 mmol) in H_2O (25 mL) and stirred at 60 °C (16 h). The solvent was removed *in vacuo* and a brown solid was obtained. The product was extracted by adding 100 mL of MeOH/CH_2Cl_2 (1:1) and stirring the mixture at room temperature for 15 minutes. The filtrate was concentrated under reduced pressure after eliminating the white KBr precipitate through filtration. The process of extraction, filtration, and concentration was repeated three times to ensure KBr removal. The resulting reddish solid was used for the next reaction. Yield 4.4 g (18.2 mmol, 90%). ^1H NMR (D_2O, 700 MHz): δ (ppm): 2.39 (3H, s, SAc), 3.18 (9H, s, NMe_3), 3.22-3.34 (2H, m), 3.41-3.50 (2H, m) (CH_2CH_2). ^{13}C NMR (D_2O, 175 MHz): δ (ppm): 21.7, 30.0, 53.1, 64.5, 199.1. ESI-MS (+, MeOH): *m*/*z* calculated for $C_7H_{16}NOS^+$: 162.0953; found: 162.0950.

Synthesis of 2-mercaptoethyl-N,N,N-trimethylammonium chloride (TMA) ligand

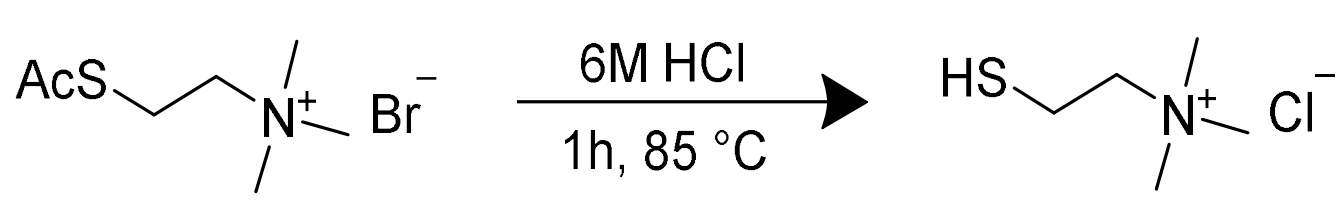

2-(Acetothioethyl)-trimethylammonium bromide (1.2 g, 5.1 mmol) stirred in 6 M HCl (10 mL) at 85 °C (1 h) before drying *in vacuo* to give a hygroscopic, pale yellow solid. Yield 732 mg (4.7 mmol, 96%). ^1H NMR (D_2O, 700 MHz): δ (ppm): 2.87-2.91 (2H, m, CH_2), 3.09 (9H, s, NMe_3), 3.47-3.51 (2H, m, CH_2). ^{13}C NMR (D_2O, 175 MHz): δ (ppm): 16.7, 53.1, 67.9. ESI-MS (+, MeOH): *m*/*z* calculated for $C_5H_{14}NS^+$: 120.0846; found: 120.0847.

Synthesis of 1-phenylpropan-2-one.

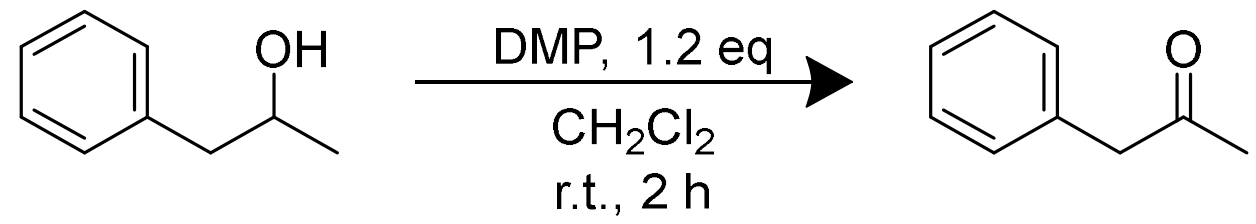

The synthesis was adapted from a reported literature procedure.[7] 1-phenylpropan-2-ol (2.0 g, 14.7 mmol) was added to Dess-Martin periodinane (7.5 g, 17.6 mmol) in anhydrous CH_2Cl_2 (30 mL) at 0 °C. The solution was allowed to warm to rt and stirred for 2 h, prior to filtration and removal of solvent in vacuo. The crude product was purified by column chromatography (SiO_2 | Hexane/Et_2O, 1:1) to afford 1-phenylpropan-2-one as a pale-yellow oil. Yield 1.7 g (12.7 mmol, 86%). ^{1}H NMR ($CDCl_3$, 700 MHz): δ (ppm): 2.15 (3H, s, CH_3), 3.70 (2H, s, CH_2), 7.22-7.23 (2H, m, phenyl H), 7.27-7.30 (1H, m, phenyl H), 7.34-7.36 (2H, m, phenyl H). ^{13}C NMR ($CDCl_3$, 175 MHz): δ (ppm): 29.1, 50.8, 126.9, 128.6, 129.3, 134.2, 206.1. ESI-MS (+, MeOH): *m*/*z* calculated for $C_9H_{11}O$: 135.0804; found: 135.0810.

Synthesis of 2-phenyl[1-^{13}C]ethanol.

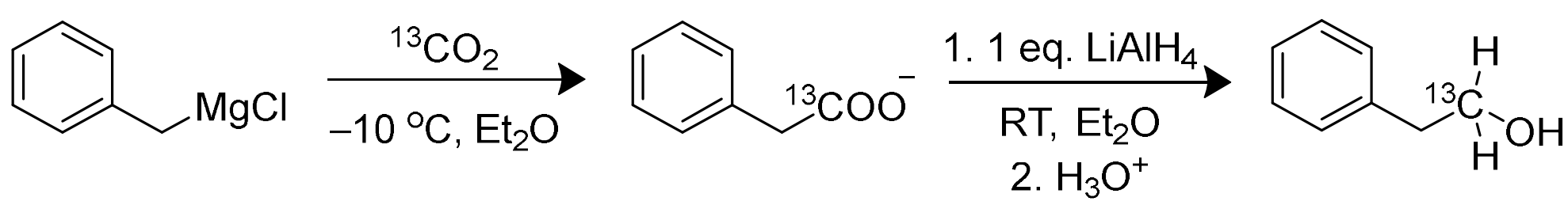

The method was adapted from literature procedures.[8–10] The requisite Grignard was first formed by refluxing benzyl chloride (8.7 mmol) in Et_2O (15 mL) over $EtBr_2$ (3-4 drops) activated Mg turnings (21 mmol) under inert conditions for 2 h. The BnMgCl Grignard solution (3 mmol) was then added into a sealed flask under $^{13}CO_2$ atmosphere (100 mL; 4.5 mmol) and allowed to stir overnight to form $Bn^{13}COO^-$. Additional Et_2O (10 mL) was added and $LiAlH_4$ (3 mmol) in Et_2O was added dropwise at −78°C and stirred overnight at ambient temperature prior to quenching (EtOAc then H_2O), drying ($MgSO_4$), filtering and removing the solvent in vacuo. Purified by flash column chromatography (SiO_2 | 5 to 60% Et_2O in hexane) to yield the liquid $Bn^{13}CH_2OH$ (179 mg, 49%). ^{1}H NMR (CD_2Cl_2, 400 MHz): δ (ppm): 2.61 (1H, br s), 2.88 (2H, q, J = 6.8 Hz), 3.83 (2H, dt, $^1J_{CH}$ = 143 Hz, $^3J_{HH}$ = 6.8 Hz), 7.40-7.25 (5H, m). ^{13}C NMR ($CDCl_3$, 700 MHz): δ (ppm): 39.2, 63.6, 126.5, 128.6, 129.1, 138.6. ESI-MS (+, MeOH): *m/z* calculated for $^{13}C^{12}C_7H_{10}O + Na^+$: 146.0663. Found: 146.0657.

Synthesis of 4-methoxy-N-octylbenzamide

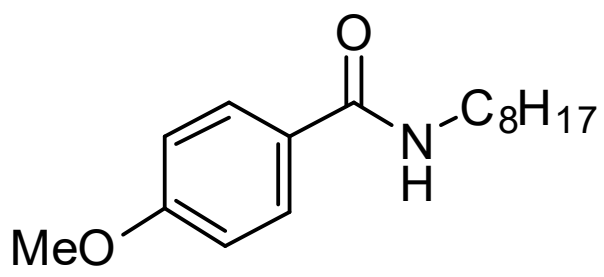

Aminocarbonylation product was batch-synthesized for analysis and comparison with the cascade reaction.[11] Ar-I (0.2 mmol, 1 equiv.), $Pd(OAc)_2$ (5 mol%), and Xantphos (10 mol%) were dissolved in dry toluene (2 mL) prior to the addition of Nu (1.0 mmol, 5 equiv.). The reaction was stirred at 25 °C for 24 h under a CO atmosphere (balloon). The solvent was then removed in vacuo. The pure product was isolated after column chromatography (SiO_2 | Hexane/Et_2O, 7:3; R_f = 0.4) as a *crystalline, pale pink solid*. Yield: 52% (27.5 mg, 0.10 mmol).

^{1}H NMR (700 MHz, $CDCl_3$): δ (ppm): 0.87 (t, *J* = 6.9 Hz, 3H), 1.28 (m *J* = 10.7, 5.9 Hz, 8H), 1.35 (p, *J* = 6.9 Hz, 2H), 1.58 (m *J* = 7.4 Hz, 2H), 3.40 (q, *J* = 6.7 Hz, 2H), 3.82 (s, 3H), 6.22 (s, 1H), 6.89 (d, *J* = 8.4 Hz, 2H), 7.72 (d, *J* = 8.5 Hz, 2H). ^{13}C NMR (176 MHz, CDCl3): δ (ppm): 14.20, 22.75, 29.33, 29.42, 29.85, 31.91, 40.20, 55.48, 113.78, 127.22, 128.74, 162.12, 167.17. ESI-MS (+, MeOH): *m/z* calculated for $C_{16}H_{26}O_2N$: 264.1958. Found: 264.1949. IR (ATR): 1180, 1240, 1463, 1493, 1532, 1606, 1625, 2848, 2917, 2962, 3333 cm^{-1}. UV-Vis (EtOAc): λ_{nm} 252, 272, 282 nm. m.p: 63-65 °C.

2D HSQC NMR experiment for Kraft lignin and calculation of theoretical monomer yield.

NMR experiments for Kraft lignin analysis were adapted from literature procedures.[12] EtOAc saturated with Kraft lignin (25 mg) was dried *in vacuo* and dissolved in DMSO-d_6 (0.6 mL). ^{1}H and ^{13}C-HSQC NMR spectra were collected on a Bruker Avance III 700 MHz spectrometer with TXO cryoprobe using the "hsqcetgpsp.2.ptg" pulse sequence.

The 2D HSQC NMR spectra were integrated for relative quantification of monomeric units and linkage motifs in Kraft lignin (**Table S4** and **Figure S11**).[13,14] Note that the maximum yield of the monomers is less than the β-O-4 fraction, as the release of a monomer requires the cleavage of two adjacent β-O-4 bonds. The volume integrals of different cross-peaks could thus be used to evaluate the theoretical monomeric yield:

Aliphatic region integrals:

β-O-4 α = 1 (1H)

β-β α = 0.69 (1H)

Aromatic region integrals:

Guaiacyl, G 2 = 16.87 (1H)

p-Hydroxyphenyl, H 2,6 = 4.17 (1H)

Total C_9 units = G + H = 21.04 per β-O-4 unit

Number of β-O-4 linkages per 100 C_9 units, P(β-O-4) = (1/21.04) × 100 = 4.75

The release of a monomer requires the cleavage of two adjacent β-O-4 bonds.

Hence, the probability of β-O-4 bonds that can be released gives the theoretical maximum monomer yield = $(P(\beta\text{-O-4}))^2$

Theoretical maximum monomer yield = 23%

Monomer isolation using preparative HPLC.

An aliquot of CdS-AET QDs was used for 10 cycles of Kraft lignin reforming with aggregation, redispersion in H_2O and addition of fresh Kraft lignin saturated EtOAc phase in each cycle. The EtOAc phase from 10 reaction cycles were combined and dried *in vacuo*.

Monomers from lignin cleavage was isolated using preparative reverse-phase high-performance liquid chromatography (Agilent 1260 Infinity with Supelco Analytical SUPELCOSIL ABZ+PLUS C18 column, 25 cm × 21.2 mm, 5 µm). Filtered solutions of the crude (diluted with H_2O, MeCN, and DMSO) were eluted with a 5–95% gradient of MeCN containing 0.05% trifluoroacetic acid over 40 min, with three fractions collected between 7 and 26 min to separate products from most of the larger polymer units. The fractions were then separated using a 5–40% gradient of the same mobile phase over 60 min, followed by solvent removal via freeze-drying (lyophilization).

Treatment of data.

All measurements were performed in triplicate, and errors were given as ± one standard deviation (σ), which was calculated as:

$$\sigma = \sqrt{\frac{\Sigma(x-\bar{x})^2}{n-1}}$$

where x is a single measured value, $\bar{x}$ is the unweighted mean value, and n is the number of repetitions.

The activity per weight of catalyst was calculated using:

$$\text{Activity } (\text{mmol}_{\text{CO or H}_2}\ \text{g}_{\text{cat}}^{-1}) = \frac{n_{H_2}\ or\ n_{CO}}{4/3\pi r_{CdS}^3 N_a \rho_{CdS} n_{QD}}$$

where n_{H_2} or n_{CO} is the amount of H_2 or CO produced (mol), r_{CdS} is the radius of the CdS QD as calculated by the TEM analysis, N_a is Avogadro's constant (6.022 × 10^{23} mol^{-1}), ρ_{CdS} is CdS density (4.84 g cm^{-3}), n_{QD} is the amount of QDs used per reaction (mol).

Supplementary Tables

Table S1. Photocatalytic CO evolution from Kraft lignin and raw biomass substrates over 24 h with CdS-AET QDs (1 μM) in 3 mL H_2O/EtOAc (1:1) at room temperature (25 °C) under an inert atmosphere (N_2). The tests were done using 447 nm LED, 120 mW cm^{-2}, unless otherwise specified, in which case, simulated solar light (AM 1.5G, 100 mW cm^{-2}) was used.

Experiment details	Substrate loading (mg mL^{-1})[a]	Solvent (v:v)	CO (μmol)	CO (mmol g^{-1}_{cat})
Kraft lignin	3.3	H_2O/EtOAc (1:1)	0.44 ± 0.01	0.88 ± 0.03
Kraft lignin (AM 1.5G)	3.3	H_2O/EtOAc (1:1)	0.396 ± 0.003	0.80 ± 0.01
Control – no lignin	-	H_2O/EtOAc (1:1)	0.037 ± 0.0001	0.074 ± 0.001
Control – no QDs	3.3	H_2O/EtOAc (1:1)	n.d.[b]	n.d.
Control – no light	3.3	H_2O/EtOAc (1:1)	n.d.	n.d.
Control – lignin + AET	3.3	H_2O/EtOAc (1:1)	0.0014 ± 0.0004	0.014 ± 0.001
Wooden branch	2.9	H_2O/EtOAc (1:1)	0.18 ± 0.02	0.36 ± 0.04
Sawdust	1.3	H_2O/EtOAc (1:1)	0.20 ± 0.01	0.41 ± 0.03
Wheatgrass	26.5	H_2O/EtOAc (1:1)	0.26 ± 0.06	0.51 ± 0.04
Bagasse	3.8	H_2O/EtOAc (1:1)	0.10 ± 0.01	0.20 ± 0.02
Rice hull	1.1	H_2O/EtOAc (1:1)	0.20 ± 0.03	0.41 ± 0.05
Cardboard	10.7	H_2O/EtOAc (1:1)	0.077 ± 0.002	0.155 ± 0.004
Coconut shell	2.7	H_2O/EtOAc (1:1)	0.10 ± 0.01	0.20 ± 0.02
Cellulose	4	H_2O/EtOAc (1:1)	0.029 ± 0.001	0.061 ± 0.001
Hemicellulose	4.2	H_2O/EtOAc (1:1)	0.032 ± 0.002	0.07 ± 0.01

[a] Substrate loading was calculated upon the saturation of Kraft lignin and raw biomass substrates in EtOAc.

[b] n.d.= not detectable.

Table S2. Optimization for the carbonylation reaction coupled with CO production from lignin using CdS QDs.[a]

Iodoanisole (µmol)	[Pd] (mol%)	Benzamide yield (µmol)	Yield (%) [b]
100	1000	0.33	75
50	1000	0.18	41
10	1000	0.03	7
1	1000	0.01	2
100	200	0.29	66
100	100	0.34	77
100	20	0.30	68
100	10	0.02	4

[a] Biphasic solvent system (H_2O/EtOAc 1:1, 3 mL) under N_2 atmosphere with aqueous layer containing CdS-AET QDs (1 µM) and EtOAc layer containing sat. Kraft lignin (3.3 mg mL^{-1}), iodoanisole, octylamine (5 equiv. of iodoanisole), $Pd(OAc)_2$, and Xantphos (2 equiv. of Pd), at 25 °C with 250 rpm orbital shaking for 24 h and LED illumination at 447 nm (120 mW cm^{-2}). [b] Yields relative to CO and determined by GC-MS.

Table S3. Effective carbon number (ECN) and yields of the major monomeric products obtained from photocatalytic Kraft lignin conversion over 24 h with CdS-AET QDs (1 μM) in 3 mL H_2O/EtOAc (1:1) at room temperature (25 °C) under an inert atmosphere (N_2).

Compound structure	ECN	Yield (mg)	Yield (wt.%)
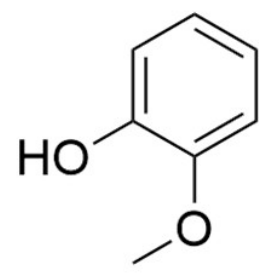	4.0	0.25 ± 0.02	5.0
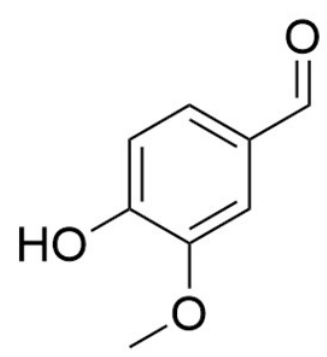	4.25	0.31 ± 0.02	6.2
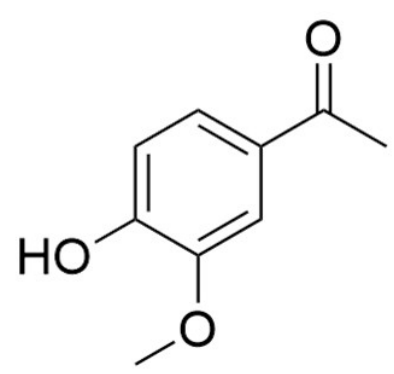	5.6	0.048 ± 0.002	0.9
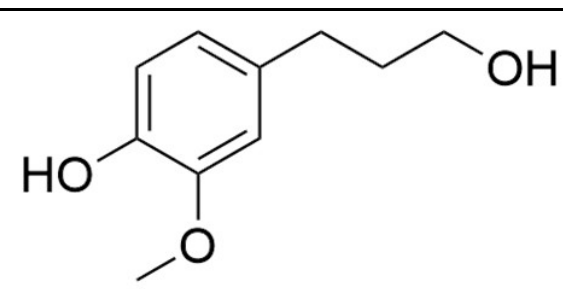	4.9	0.18 ± 0.01	3.6
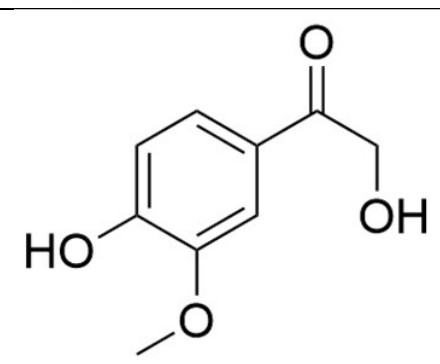	3.5	0.14 ± 0.01	2.8
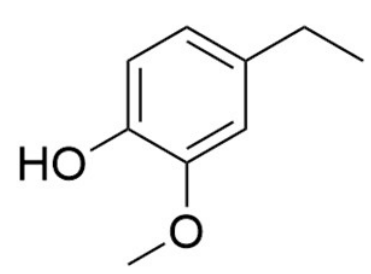	6.0	0.0045 ± 0.0001	0.09

Table S4. ^1H-^{13}C cross signal assignments for the identification and quantification of different linkages and aromatic monomers in Kraft lignin.

Label	δH/δC (ppm)	Assignments
β-O-4 (α)	4.75/71.5	$C_α$-$H_α$ in β-O-4 unit
β-O-4 (β)	4.28/84.22	$C_β$-$H_β$ in β-O-4 unit
β-β (α)	4.59/85.78	$C_α$-$H_α$ in β-β resinol unit
β-β (β)	3.01/53.97	$C_β$-$H_β$ in β-β resinol unit
β-β (γ)	4.10/61.92	$C_γ$-$H_γ$ in β-β resinol unit
G_2	7.11/109.96	C_2-H_2 in guaiacyl unit (G)
$H_{2,6}$	6.92/126.21	$C_{2,6}$-$H_{2,6}$ in p-hydroxyphenyl unit (H)

Table S5. Yields of the major monomeric products obtained using recycled CdS-AET QDs (1 μM) for photocatalytic Kraft lignin conversion over 24 h in 3 mL H_2O/EtOAc (1:1) at room temperature (25 °C) under an inert atmosphere (N_2).

Monomer structure	Yield (wt.%) per cycle									
	1	2	3	4	5	6	7	8	9	10
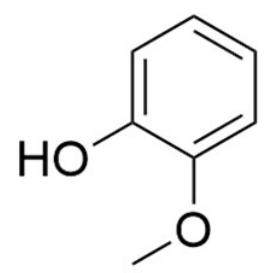	5.0	5.7	4.4	5.0	4.8	4.7	4.8	4.7	5.0	4.9
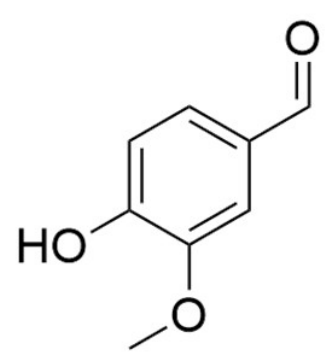	6.2	5.7	5.2	6.1	6.0	6.1	5.9	6.1	5.6	5.4
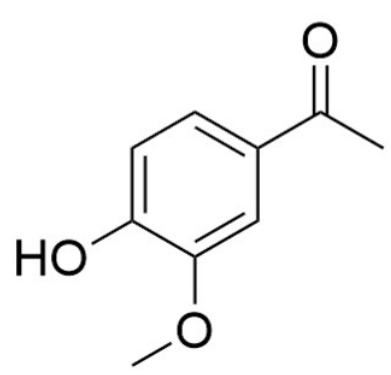	0.9	1.3	0.8	1.1	1.1	1.1	1.0	1.0	1.1	1.1
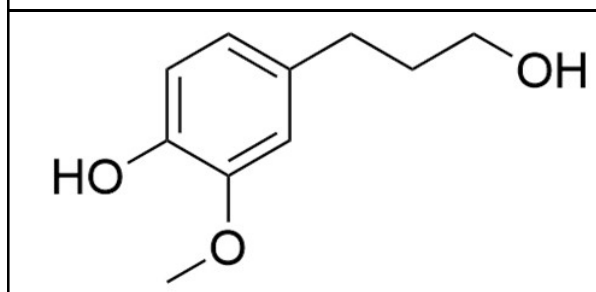	3.6	3.5	3.0	3.8	3.7	3.9	3.6	3.7	3.6	3.4
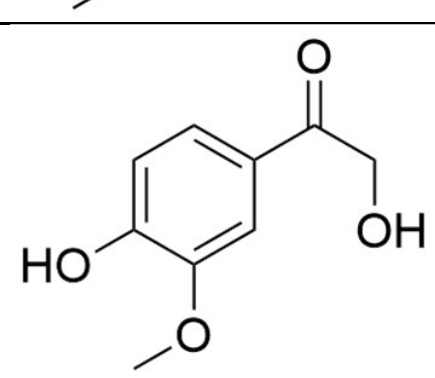	2.8	1.7	1.5	1.9	1.8	1.8	2.0	1.9	2.1	2.0
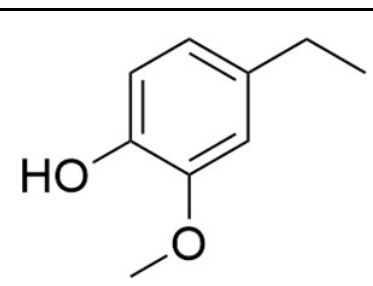	0.09	0.1	0.08	0.1	0.09	0.1	0.1	0.1	0.1	0.1

Table S6. β-O-4 cleavage products from photocatalytic lignin model conversion (447 nm, 120 mW cm^{-2}) over 24 h with CdS-AET QDs (1 μM) in 3 mL H_2O/MeCN (2:1) or H_2O/EtOAc (1:1) at room temperature (25 °C) under an inert atmosphere (N_2).

Entry	Reactant	Medium	Conversion	Yield
1	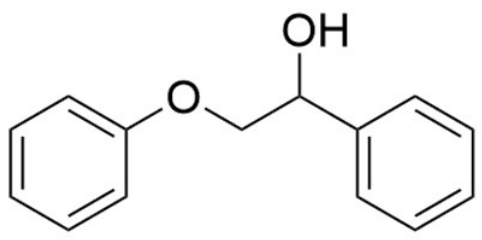	H_2O/ MeCN (2:1)	>95%	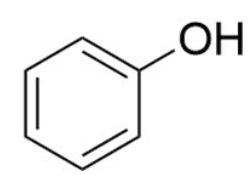 (89%) 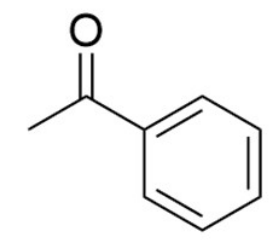 (75%)
2	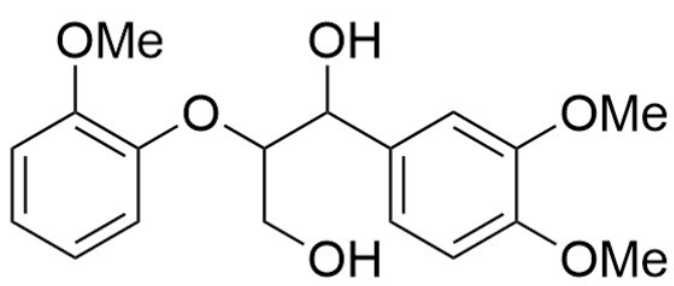	H_2O/ MeCN (2:1)	>95%	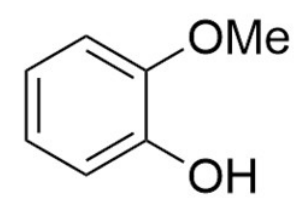 (98%) 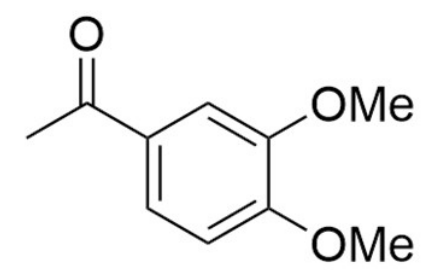 (3%) 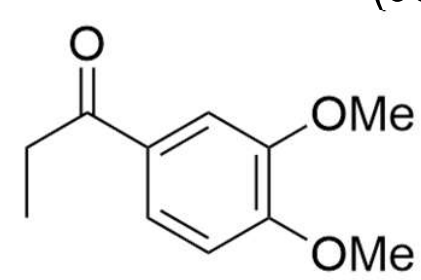 (23%) 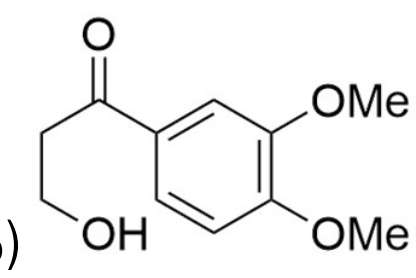 (22%) 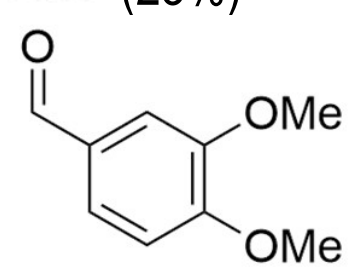 (1%)
3	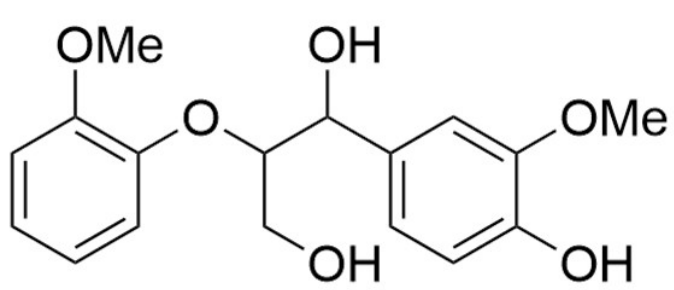	H_2O/ MeCN (2:1)	>95%	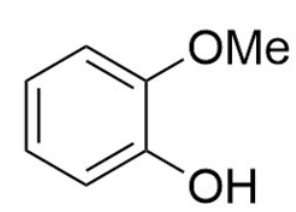 (97%) 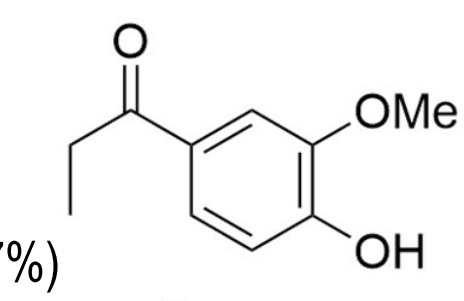 (15%) 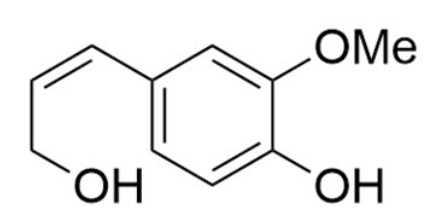 (8%) 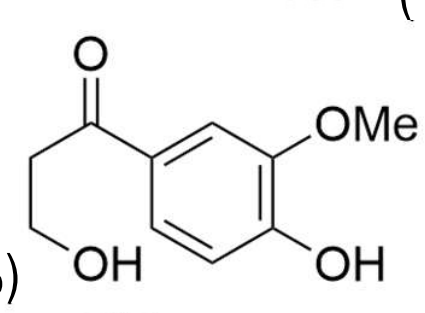 (15%) 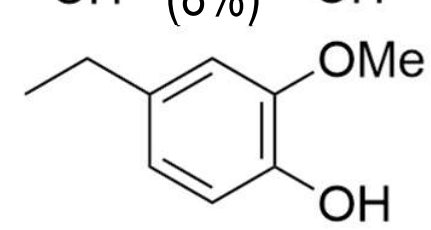 (1%)
4	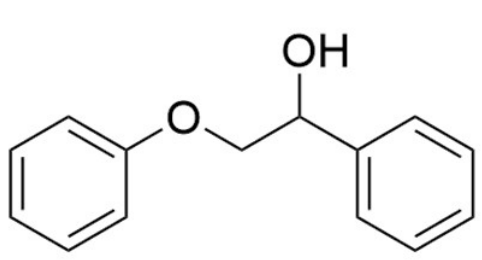	H_2O/EtOAc (1:1)	90%	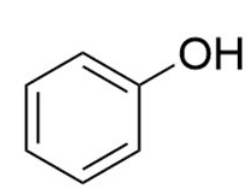 (82%) 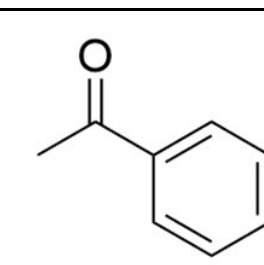 (70%)

5	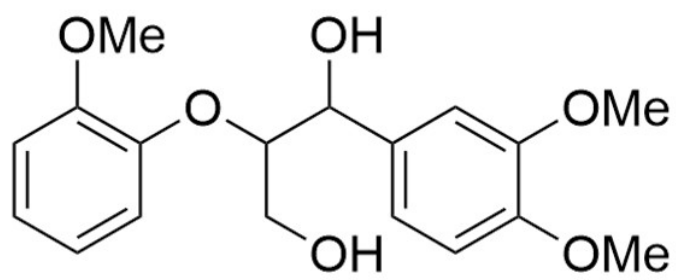	H_2O/EtOAc (1:1)	82%	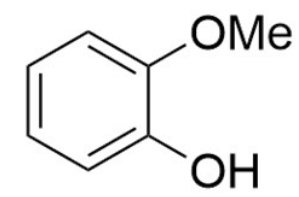 (74%) 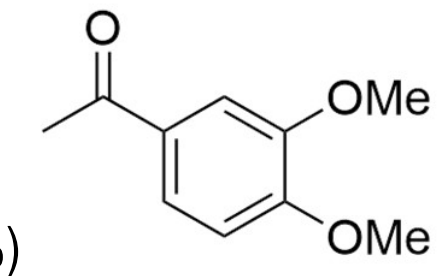 (2%) 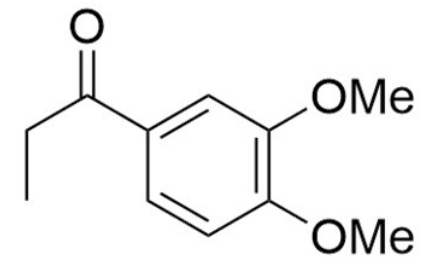 (19%) 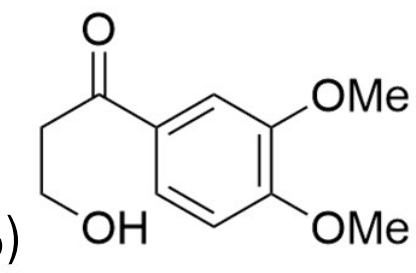 (17%) 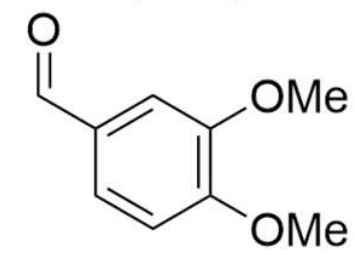 (1%)
6	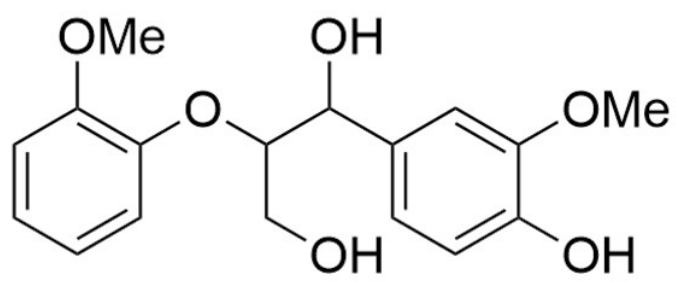	H_2O/EtOAc (1:1)	20%	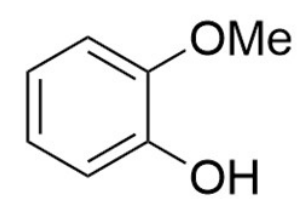 (24%) 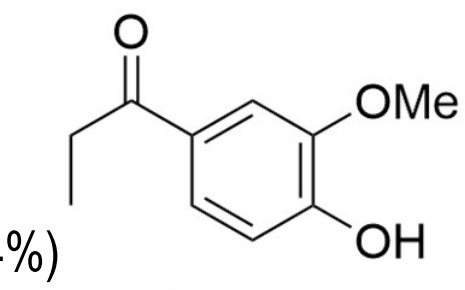 (3%) 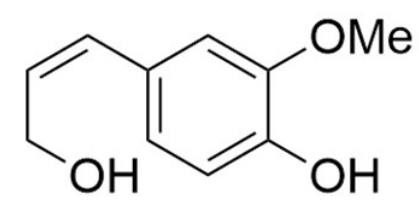 (7%) 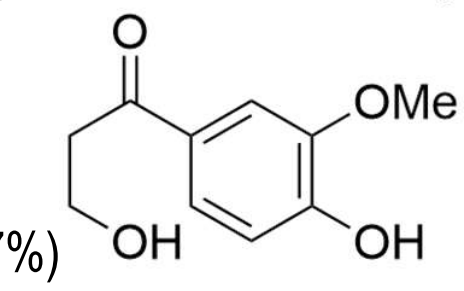 (7%)

Table S7. Comparison of catalytic performances for lignin model reforming.

Catalyst	Substrate	Reaction condition	Conversion	Major products	Activity ($mmolg^{-1}_{cat}$)	Ref.
CdS	**1a**	RT, visible light, 3 h	> 99%	AP + PhOH	9.3	14
$ZnIn_2S_4$	**1a**	60 °C, visible light, 24 h	82%	AP + PhOH	11.5	15
p-CN/P-PDI_3	**1a**	RT, visible light, O_2, 3 h	99%	BA/PF	0.6	16
mpg-C_3N_4	**1a**	RT, 450 nm, O_2, 10h	32%	BA/PF	1.6	17
Pd/$ZnIn_2S_4$+TiO_2	**1a**	42 °C, 455 nm, 4 h	99%	AP + PhOH	19.8	18
Ni/CdS	**1a**	RT, 440 nm, 3 h	99%	AP + PhOH	9.9	19
$Cu(OAc)_2$-DMSO	**1a**	80 ºC, 0.4 MPa O_2, 6 h, KOH	99%	BA	47.6	20
1. $VOSO_4$<br>2. $Cu(OAc)_2$-ligand	**1a**	1. TEMPO, 0.4 MPa O_2, 100 ºC, 24 h<br>2. 0.4 MPa O_2, 80 ºC, 6 h	(76%)<br>96%	PhOH + BA	18.42	21
CdS	**1b**	RT, visible light, 3 h	95%	Guaiacol	8.9	14
HTc-Cu-V	**1b**	135 °C, 0.6 MPa O_2, pyridine, 16 h	95%	VA + VOOH	14.2	22
$[Ru(Cl)(H)(PPh_3)_3]$/triphos	**1b**	160 °C, xylene, 16 h	77%	VA	9.1	23
CdS	**1a**	RT, 447 nm, 24 h	> 95%	AP + PhOH	14.25	This work
CdS	**1b**	RT, 447 nm, 24 h	>98%	Guaiacol	14.7	

1a: 2-phenoxy-1-phenylethanol; AP: acetophenone; PhOH: phenol; BA: benzaldehyde; PF: phenyl formate; **1b**: 1-(3,4-dimethoxyphenyl)-2-(2-methoxyphenoxy)-1,3-propanediol; VA: veratraldehyde; VOOH: veratric acid.

Table S8. β-O-4 cleavage products from photocatalytic lignin model conversion (447 nm, 120 mW cm^{-2}) over 24 h with oleic acid-capped CdS QDs (CdS-OA) and CdS-AET QDs (1 μM) in 3 mL solvent at room temperature (25 °C) under an inert atmosphere (N_2).[a]

Experiment details	Photocatalyst	Reactant	Solvent (v:v)	Conversion	Yield
Without ligand exchange	CdS-OA	**1a**	MeCN	<1%	AP (n.d.) + PhOH (0.2%)
	CdS-OA	**1a**	H_2O/MeCN (2:1)	<5%	AP (2%) + PhOH (2%)
Model compound **1a** (5 mM)	CdS-AET	**1a**	H_2O/MeCN (2:1)	>95%	AP (75%) + PhOH (89%)
Model compound **1a** (5 mM)	Decapped CdS	**1a**	H_2O/MeCN (2:1)	>95%	AP (85%) + PhOH (95%)
Control- no QDs[b]	-	**1a**	H_2O/MeCN (2:1)	n.d.	n.d.
Control- no light[b]	CdS-AET	**1a**	H_2O/MeCN (2:1)	n.d.	n.d.

[a] **1a**: 2-phenoxy-1-phenylethanol; AP: acetophenone; PhOH: phenol. n.d.= not detectable. [b] 5 mM model compound **1a** was used as the substrate for control experiments.

Table S9. Photocatalytic CO evolution and lignin model β-O-4 cleavage product generation (447 nm, 120 mW cm^{-2}) over 24 h with CdS-AET QDs (1 μM) in 3 mL H_2O/MeCN (2:1) at room temperature (25 °C) under an inert atmosphere (N_2). [a]

Experiment details	Solvent (v:v)	Time (h)	CO (μmol)	Phenol (μmol)	Acetophenone (μmol)	Guaiacol (μmol)
Model compound **1a** (5 mM)	H_2O/MeCN (2:1)	0<br>2<br>4<br>8<br>16<br>24	0.00 ± 0.00<br>0.001 ± 0.001<br>0.004 ± 0.001<br>0.015 ± 0.004<br>0.016 ± 0.002<br>0.045 ± 0.007	0.00 ± 0.00<br>1.5 ± 0.1<br>3.1 ± 0.2<br>10 ± 2<br>13.1 ± 0.2<br>13.3 ± 0.1	0.00 ± 0.00<br>1.38 ± 0.02<br>2.9 ± 0.1<br>9 ± 2<br>10.8 ± 0.2<br>11.1 ± 0.1	-
Model compound **1b** (5 mM)	H_2O/MeCN (2:1)	0<br>2<br>4<br>8<br>16<br>24	0.00 ± 0.00<br>0.005 ± 0.001<br>0.02 ± 0.01<br>0.04 ± 0.01<br>0.08 ± 0.02<br>0.11 ± 0.03	-	-	0.00 ± 0.00<br>1.6 ± 0.1<br>7.77 ± 0.04<br>10.7 ± 0.5<br>14 ± 2<br>14 ± 1
Model compound **1c** (5 mM)	H_2O/MeCN (2:1)	0<br>2<br>4<br>8<br>16<br>24	0.00 ± 0.00<br>0.005 ± 0.001<br>0.012 ± 0.006<br>0.02 ± 0.01<br>0.05 ± 0.01<br>0.06 ± 0.01	-	-	0.00 ± 0.00<br>4.9 ± 0.2<br>9.4 ± 0.1<br>12.6 ± 0.8<br>16 ± 2<br>16 ± 1
Control – no substrate	H_2O/MeCN (2:1)	24	n.d.	-	-	-
Control – no QDs[b]	H_2O/MeCN (2:1)	24	n.d.	n.d.	n.d.	-
Control – no light[b]	H_2O/MeCN (2:1)	24	n.d.	n.d.	n.d.	-
Kraft lignin (3.3 mg mL^{-1})	H_2O/MeCN (2:1)	24	n.d.	-	-	-

[a] **1a**: 2-phenoxy-1-phenylethanol; **1b**: 1-(3,4-dimethoxyphenyl)-2-(2-methoxyphenoxy)-1,3-propanediol; **1c**: guaiacyl glycerol-β-guaiacyl ether. [b] 5 mM model compound **1a** was used as the substrate for control experiments. n.d.= not detectable.

Table S10. Control experiments with different scavengers and reactants for the mechanistic study of CO evolution over 24 h with CdS-AET QDs (1 µM) in 3 mL H_2O/EtOAc (1:1) at room temperature (25 °C) under an inert atmosphere (N_2). Liquid chromatography-mass spectrometry (LCMS) was used to detect the 5,5-dimethyl-1-pyrroline N-oxide (DMPO) radical adducts.

**Reactant (0.5M)**	**Time (h)**	**Scavenger**	**CO (µmol)**	**H_2 (µmol)**	**Liquid product (µmol)**
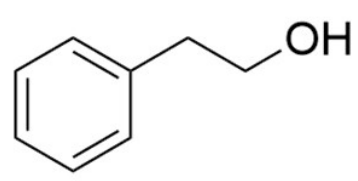	3 h<br>24 h	-	0.027 ± 0.002<br>0.13 ± 0.02	0.001 ± 0.001<br>0.58 ± 0.04	0.03 ± 0.001<br>0.4 ± 0.1<br>(Toluene)
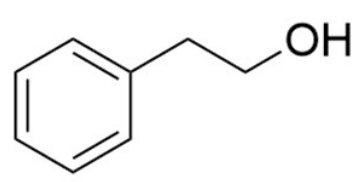	3 h	Electron, 2 mM $K_2S_2O_8$	0.012 ± 0.004	0.006 ± 0.004	n.d.[a]
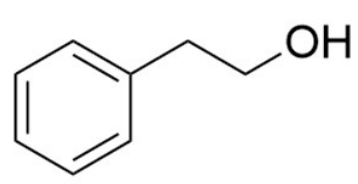	3 h	Hole, 4 mM Na_2S + 2 mM Na_2SO_3	0.005 ± 0.002	0.8 ± 0.3	n.d.
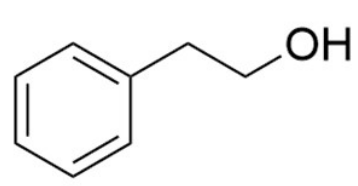	3 h	Radical, 25 mM DMPO (detection LCMS)	0.021 ± 0.002	0.003 ± 0.004	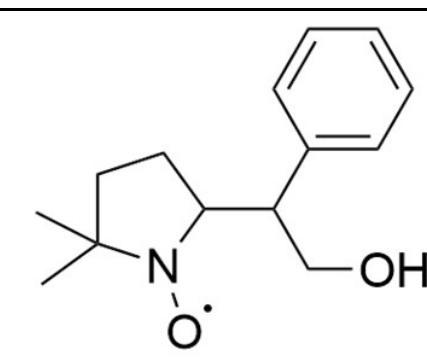<br>Calc [M^+]: 234.1494,<br>found: 234.1505
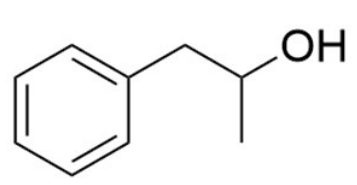	24 h	-	0.055 ± 0.003	0.52 ± 0.03	0.025 ± 0.001<br>(Toluene)
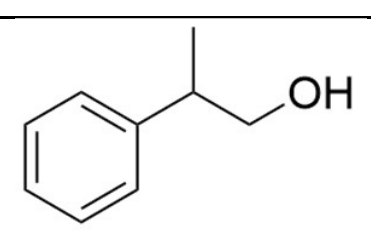	24 h	-	0.152 ± 0.004	2.15 ± 0.05	0.055 ± 0.004<br>(Ethylbenzene)
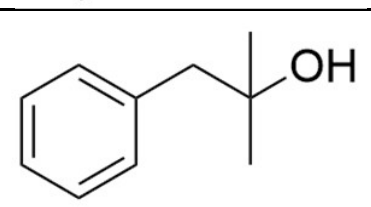	24 h	-	0.011 ± 0.007	1.5 ± 0.2	n.d.
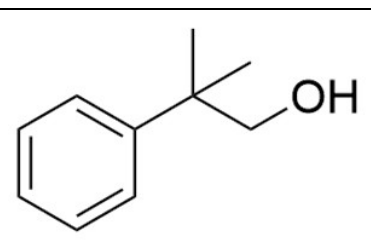	24 h	-	0.041 ± 0.006	0.17 ± 0.02	n.d.
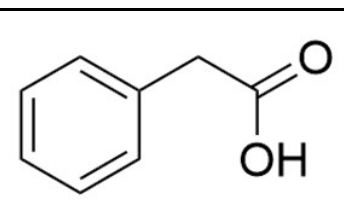	24 h	-	0.031 ± 0.001	0.6 ± 0.3	0.67 ± 0.07<br>(Toluene)
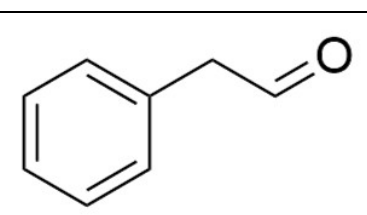	1 h<br>24 h	-	0.34 ± 0.03<br>61 ± 4	n.d.<br>1.4 ± 0.2	0.76 ± 0.03<br>85 ± 9<br>(Toluene)
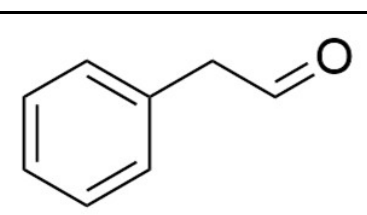	1 h	Electron, 10 mM $K_2S_2O_8$	0.11 ± 0.03	n.d.	0.609 ± 0.004<br>(Toluene)
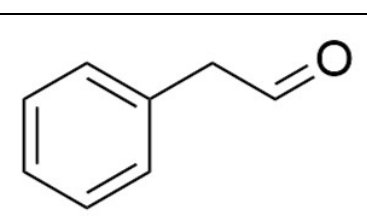	1 h	Hole, 4 mM Na_2S + 2 mM Na_2SO_3	1.16 ± 0.04	n.d.	1.32 ± 0.01<br>(Toluene)

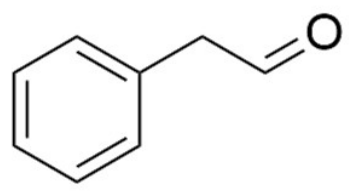	1 h	Radical, 25 mM DMPO (detection LCMS)	0.161 ± 0.006	n.d.	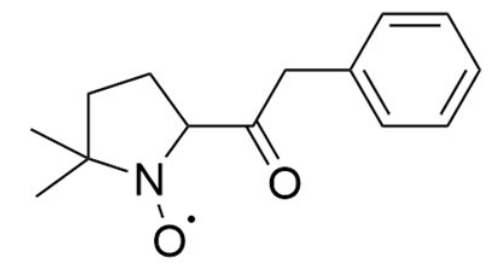 Calc [M+]: 232.1338, found: 232.1330 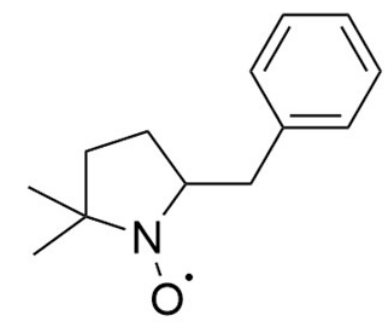 Calc [M+]: 204.1388, found: 204.1385
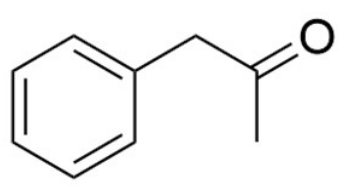	24 h	-	0.15 ± 0.01	0.038 ± 0.001	0.25 ± 0.01 (Toluene)
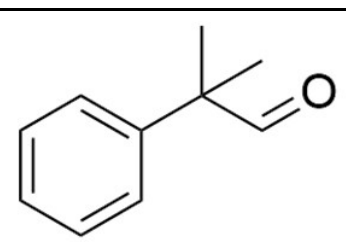	24 h	-	30 ± 1	0.5 ± 0.2	4.7 ± 0.2 (Isopropylbenzene)
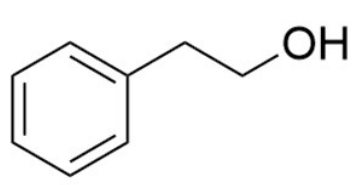 [b]	110 h	-	0.42 ± 0.1 (3% yield)	0.32 ± 0.02	0.73 ± 0.08 (Toluene)
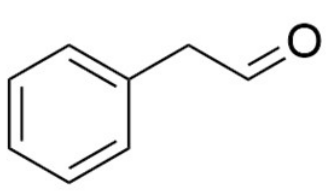 [b]	72 h	-	9.7 ± 0.8 (65% yield)	0.48 ± 0.04	12 ± 4 (Toluene)

[a] n.d. = not detectable. [b] CO evolution over 2-phenylethanol (10 mM) and phenylacetaldehyde (10 mM) was studied with CdS-AET QDs (5 μM) with other reaction parameters unchanged.

Supplementary Figures

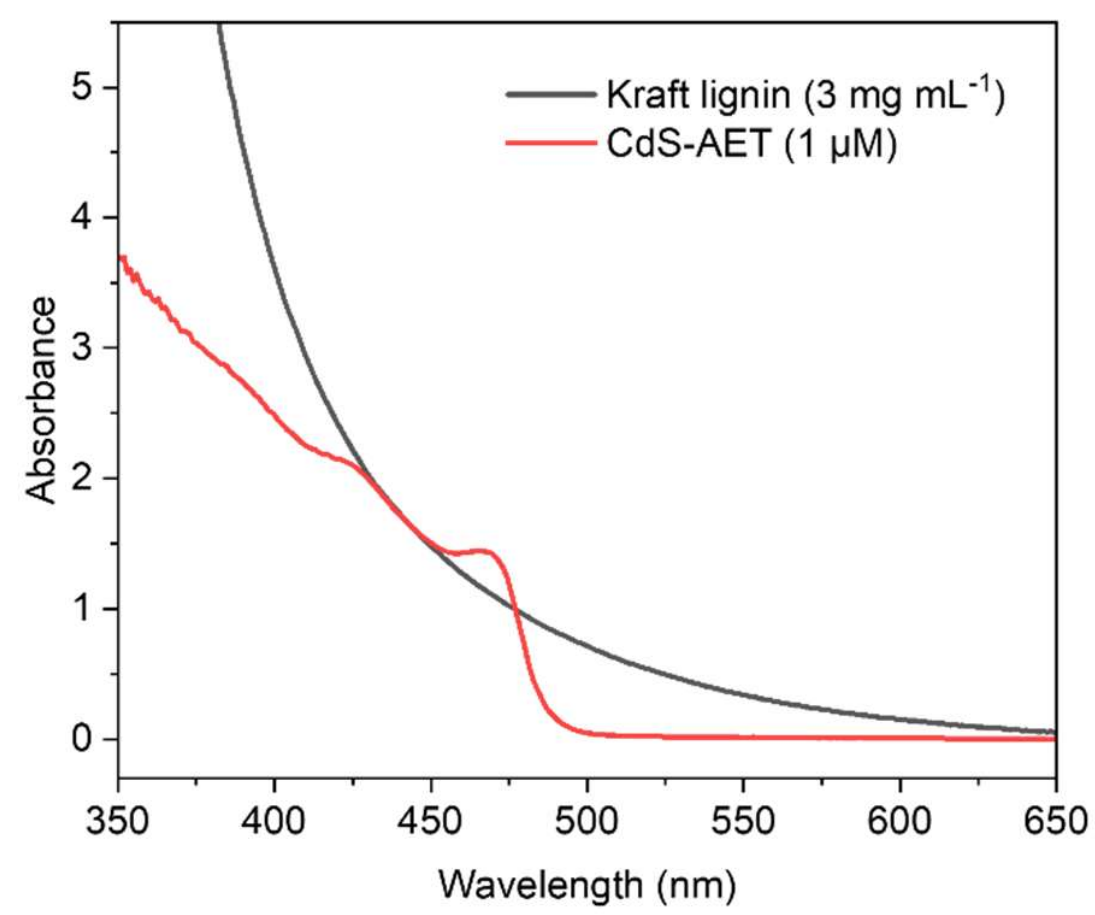

Figure S1: Absorption spectra of Kraft lignin (3 mg mL^{-1}) and CdS-AET QDs (1 μM) in H_2O/MeCN (2:1) monophasic system.

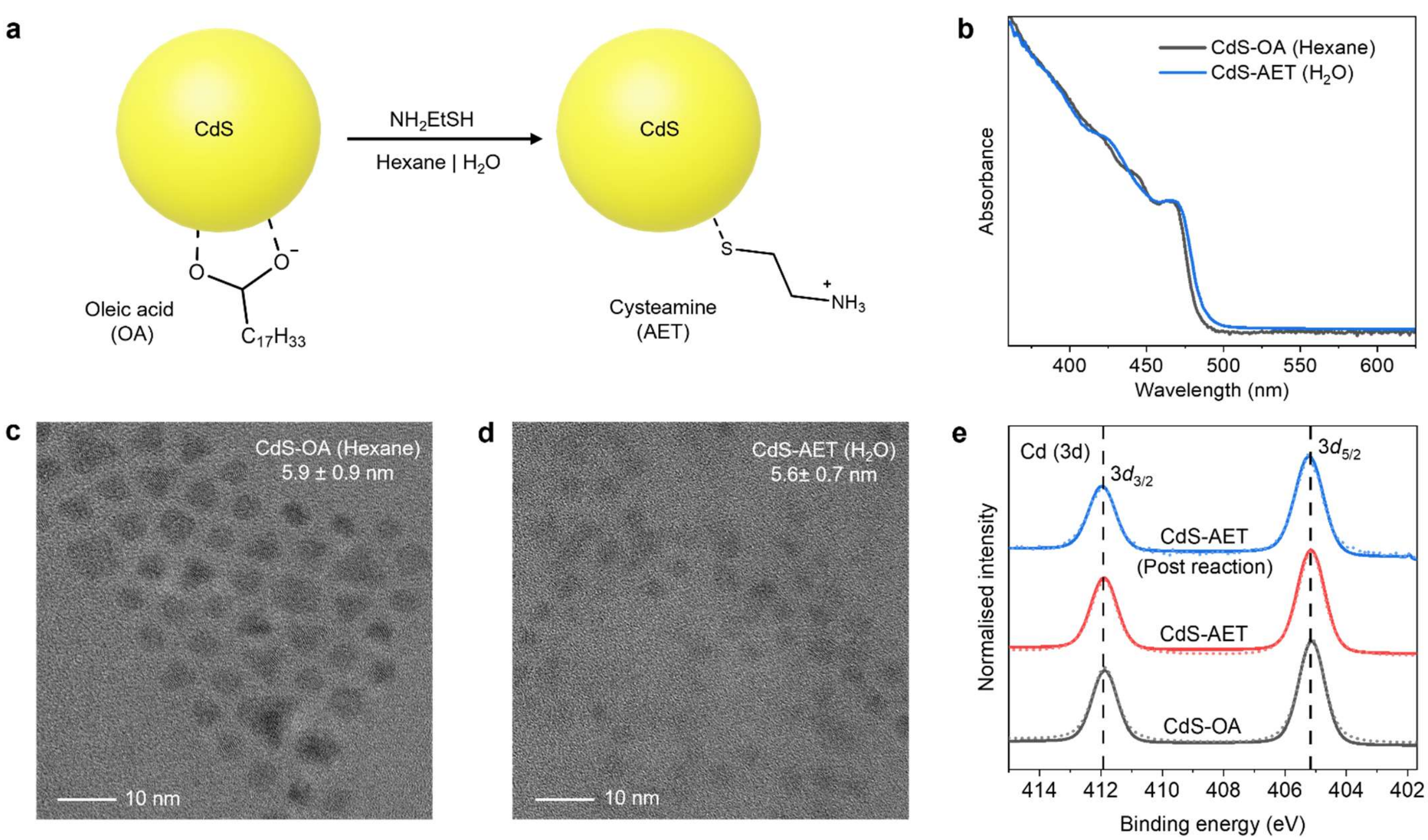

Figure S2: Characterization of CdS QDs capped with oleic acid (OA) and 2-aminoethanethiol (AET) in hexane and H_2O, respectively. a, Schematic of the ligand exchange procedure for the dispersion of CdS QDs in H_2O. **b**, Normalized UV-Vis absorption spectra of CdS-OA and CdS-AET. **c**, **d**, TEM image of CdS-OA and CdS-AET. **e**, XPS spectra of the Cd (3d) region of CdS QDs with OA and AET capping after isolation from hexane and water, respectively, and CdS-AET QDs after 24 h photocatalysis reaction.

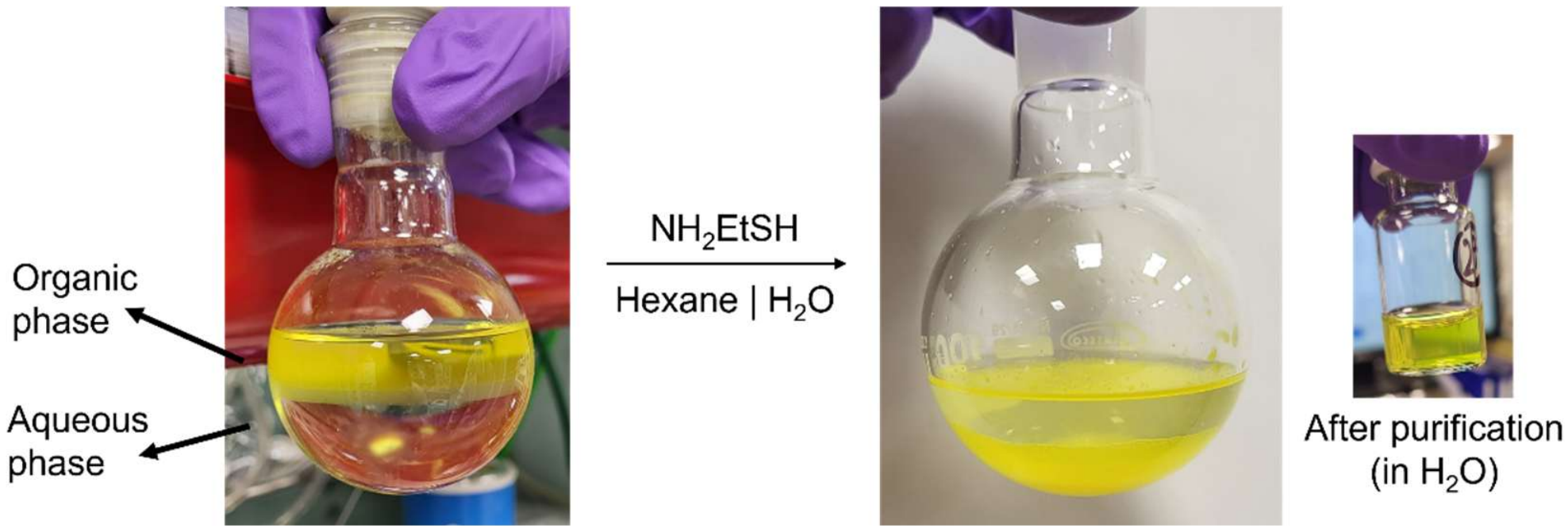

Figure S3: Ligand exchange procedure for the phase transfer of CdS-OA QDs from hexane to CdS-AET QDs in H_2O. A clear suspension of CdS-AET in H_2O was obtained after washing.

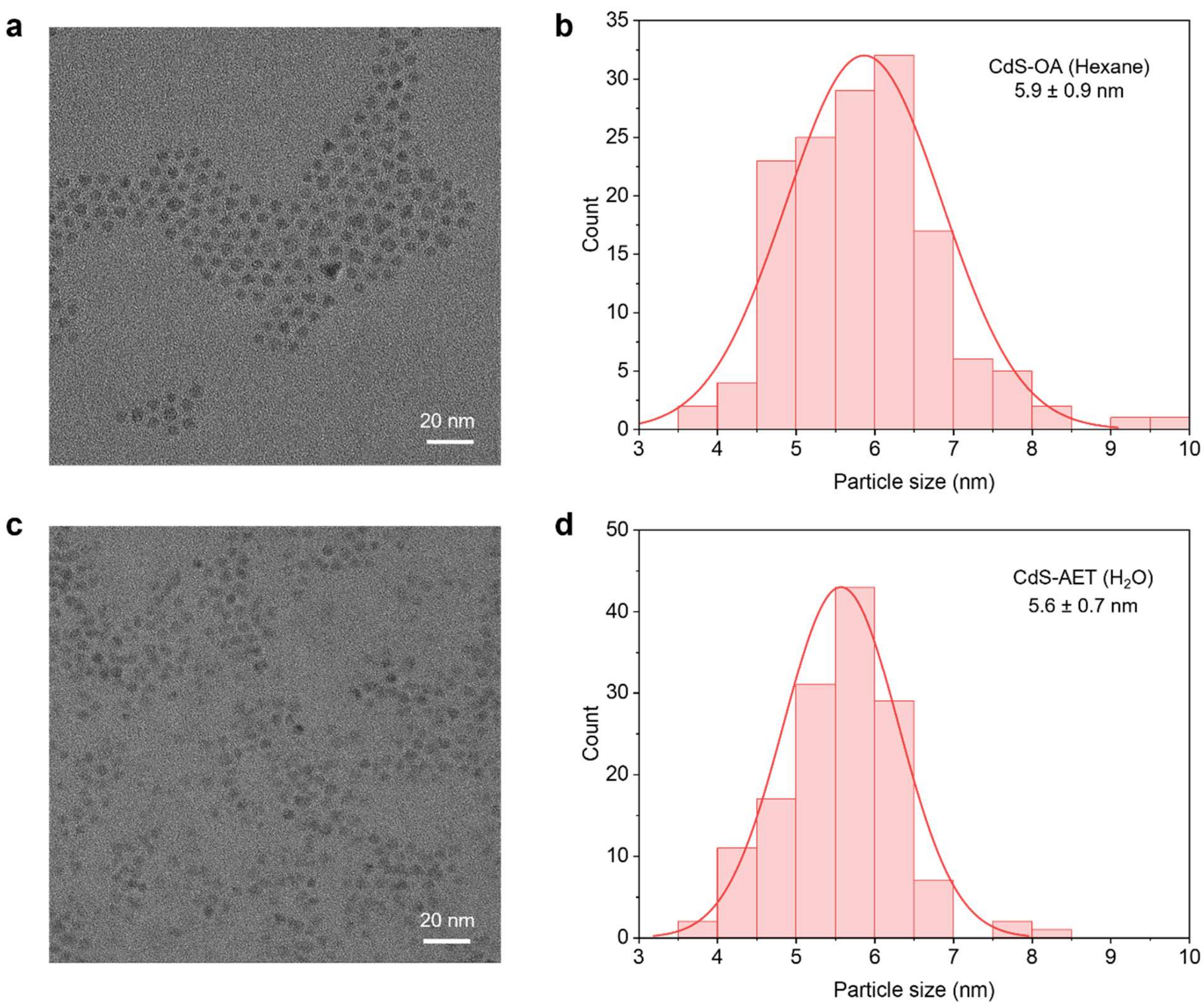

Figure S4: a,b, TEM image of CdS-OA QDs in hexane (**a**) and the corresponding size distribution (**b**). **c,d,** TEM image of CdS-AET QDs in water (**c**) and the corresponding size distribution (**d**).

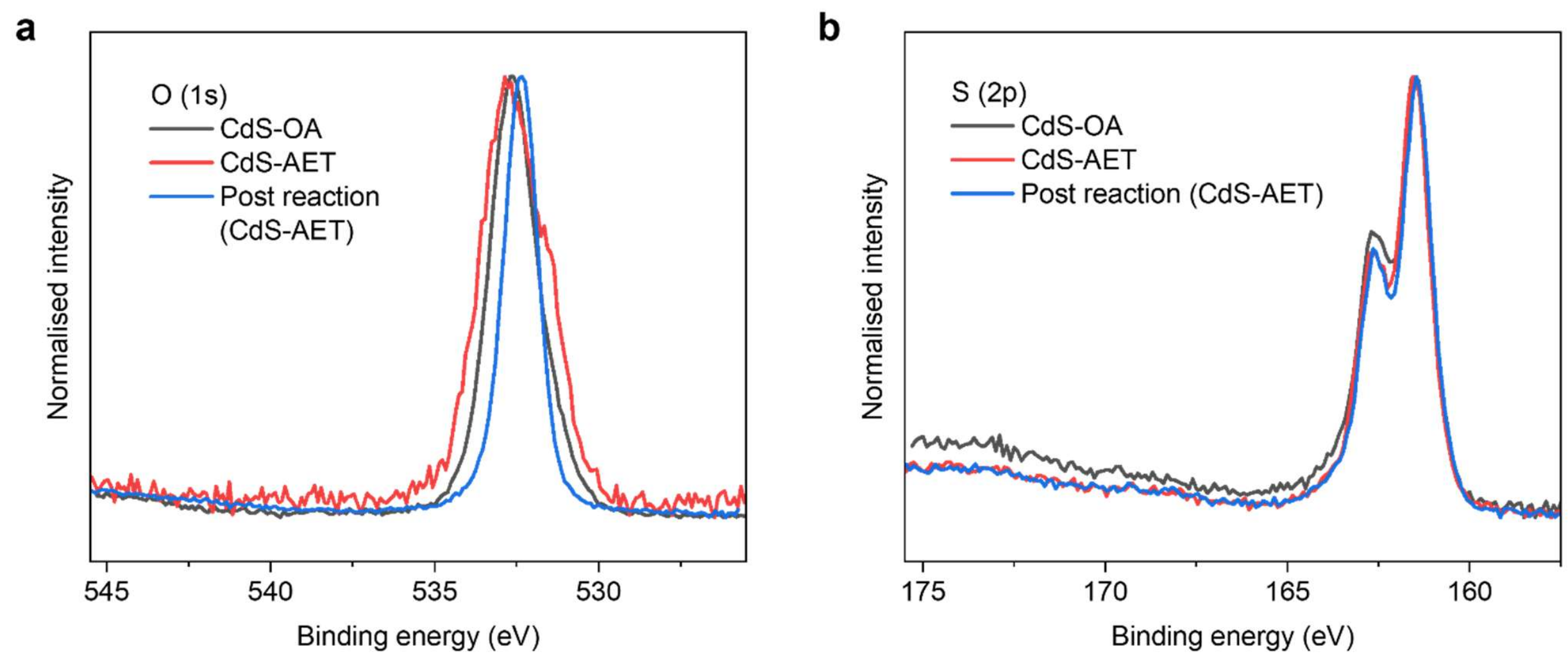

Figure S5: XPS spectra of CdS QDs with OA and AET capping after isolation from hexane and water, respectively, and CdS-AET QDs after 24h photocatalysis reaction in the region of **(a)** O (1s) and **(b)** S (2p).

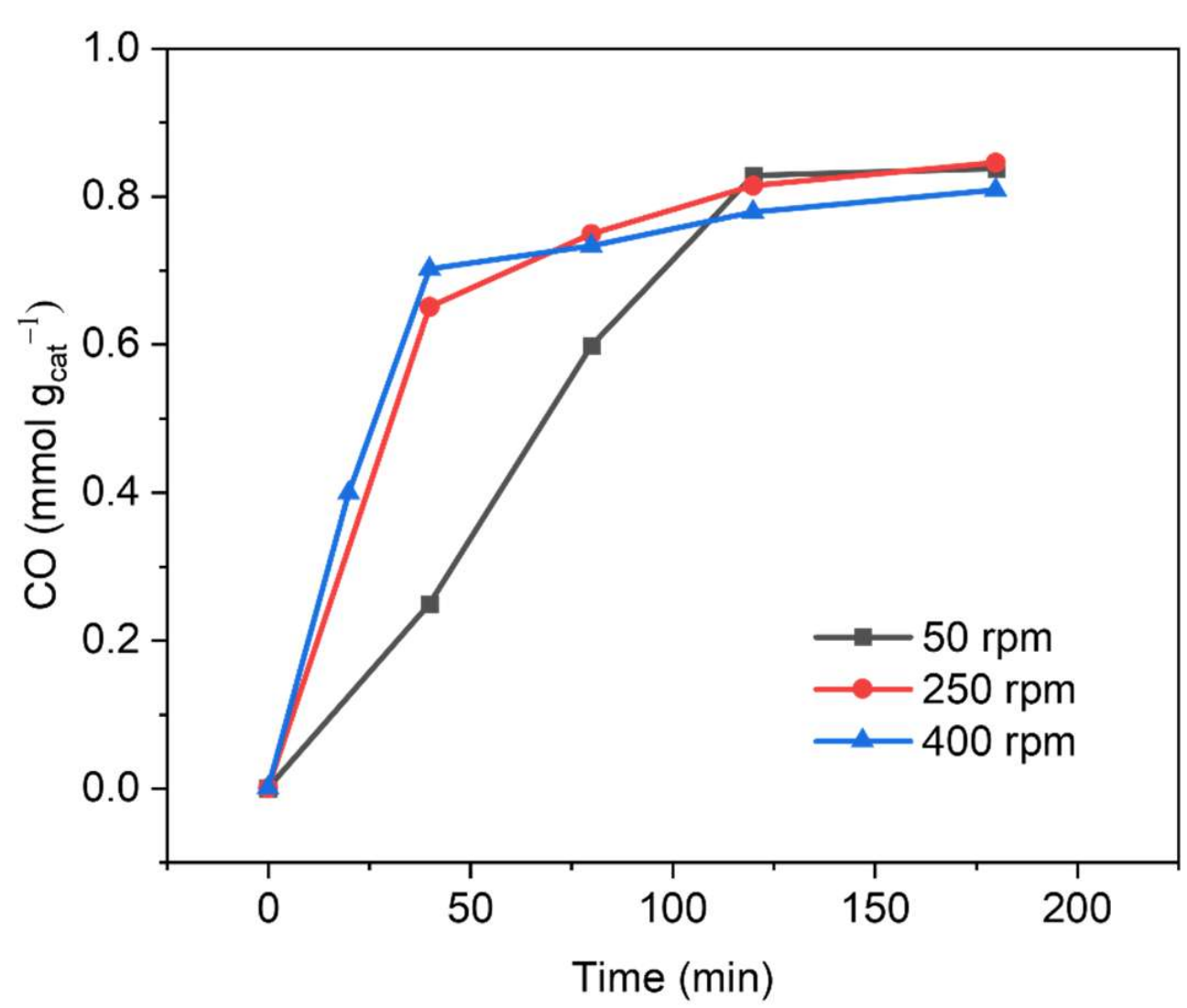

Figure S6: Kinetic trace of photocatalytic CO evolution from Kraft lignin using CdS-AET QDs (1 μM) in water below a Kraft lignin-saturated (3.3 mg mL^{-1}) EtOAc layer in a biphasic solvent system (3 mL, H_2O/EtOAc 1:1). All measurements with 447 nm, 120 mW cm^{-2}, illumination at 25 °C under N_2 atmosphere with orbital shaking.

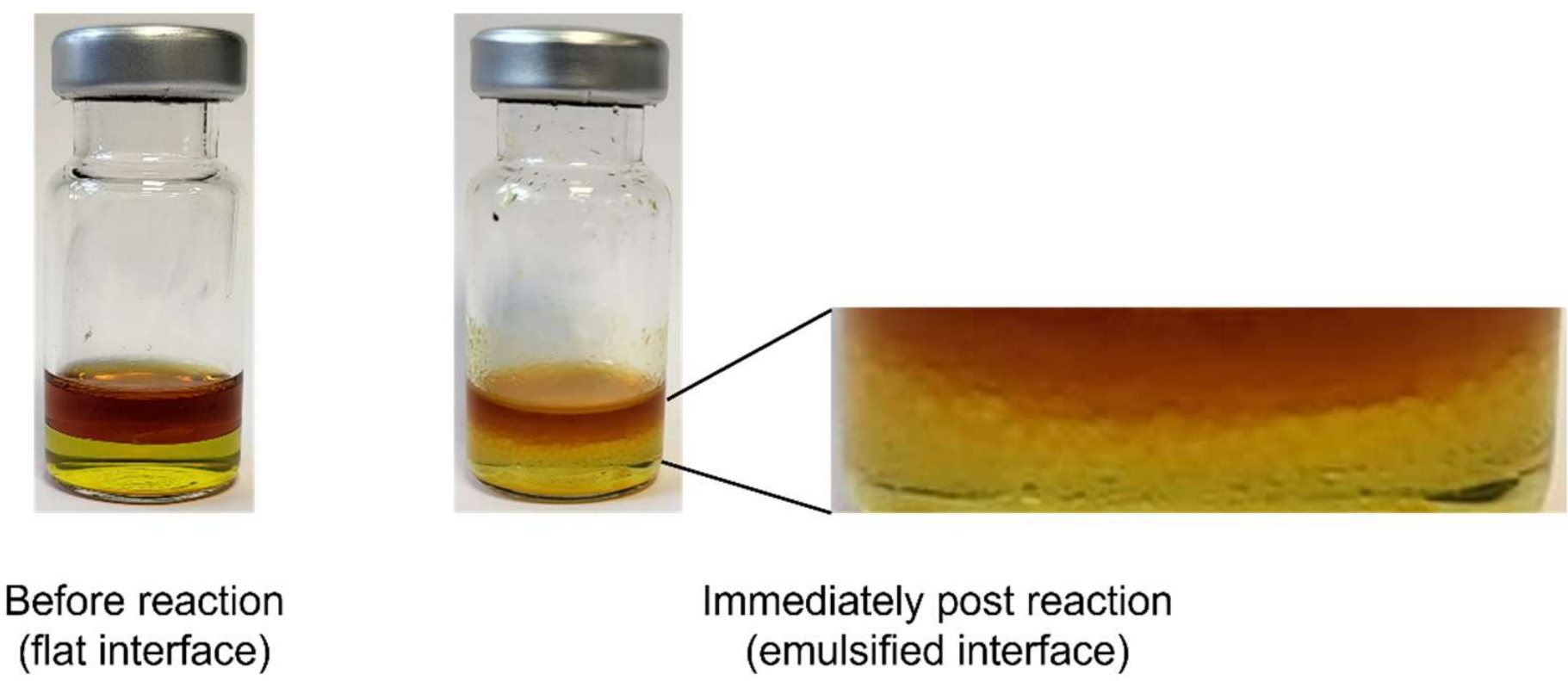

Figure S7: Bilayer system for CO generation from Kraft lignin before and after reaction.

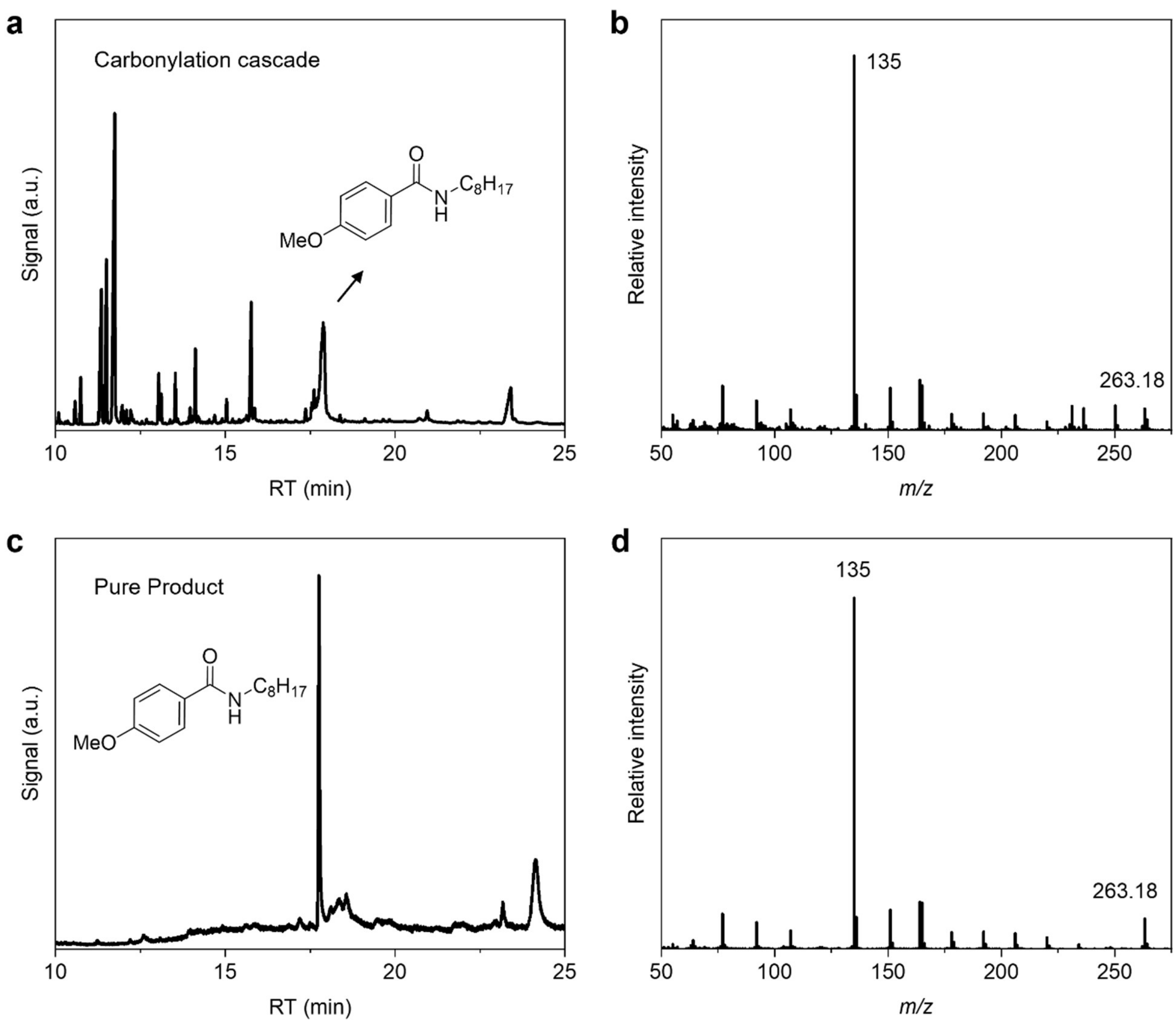

Figure S8: GC spectra and the corresponding mass spectra of the crude reaction mixture from aminocarbonylation cascade in lignin-saturated EtOAc (**a**, **b**) and the pure product (**c**,**d**).

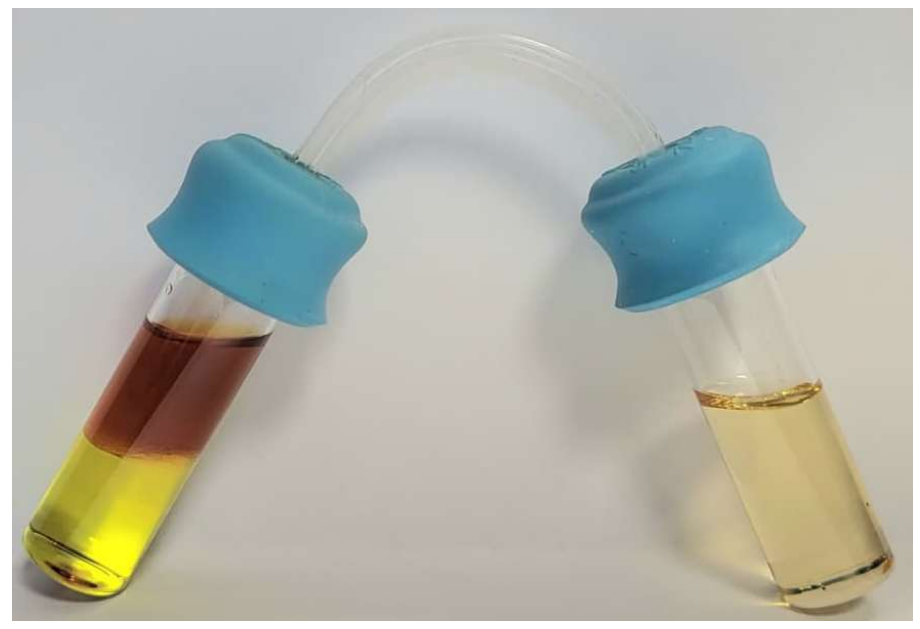

Figure S9: Two-compartment setup (7.5 mL) for carbonylation reaction from CO generated via Kraft lignin conversion using CdS-AET QDs.

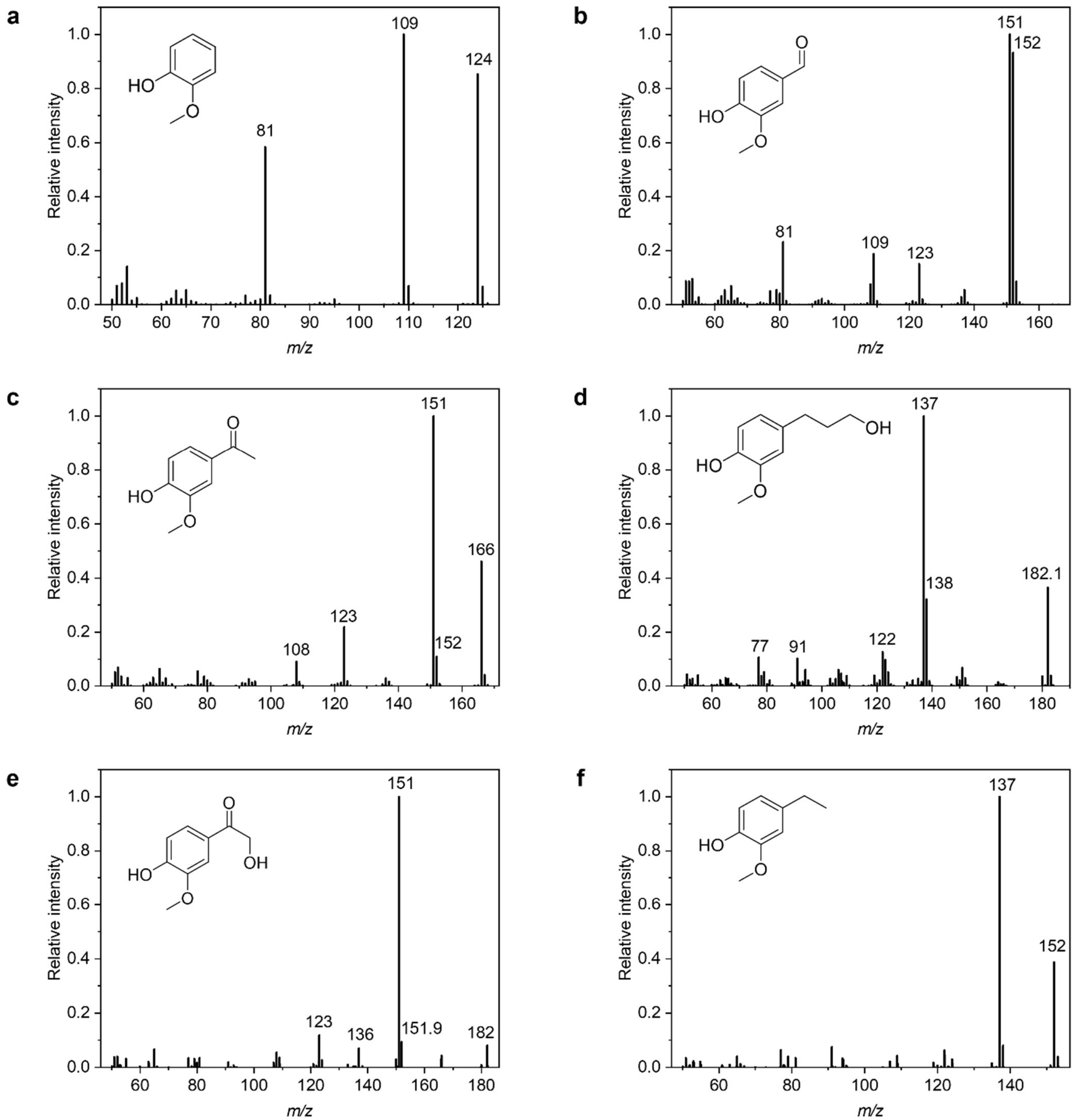

Figure S10: a, **b, c, d, e, f,** Mass spectra of the monomers obtained from photocatalytic Kraft lignin conversion. The insets show the respective monomer compound structures obtained via GC-MS and confirmed by LC-MS.

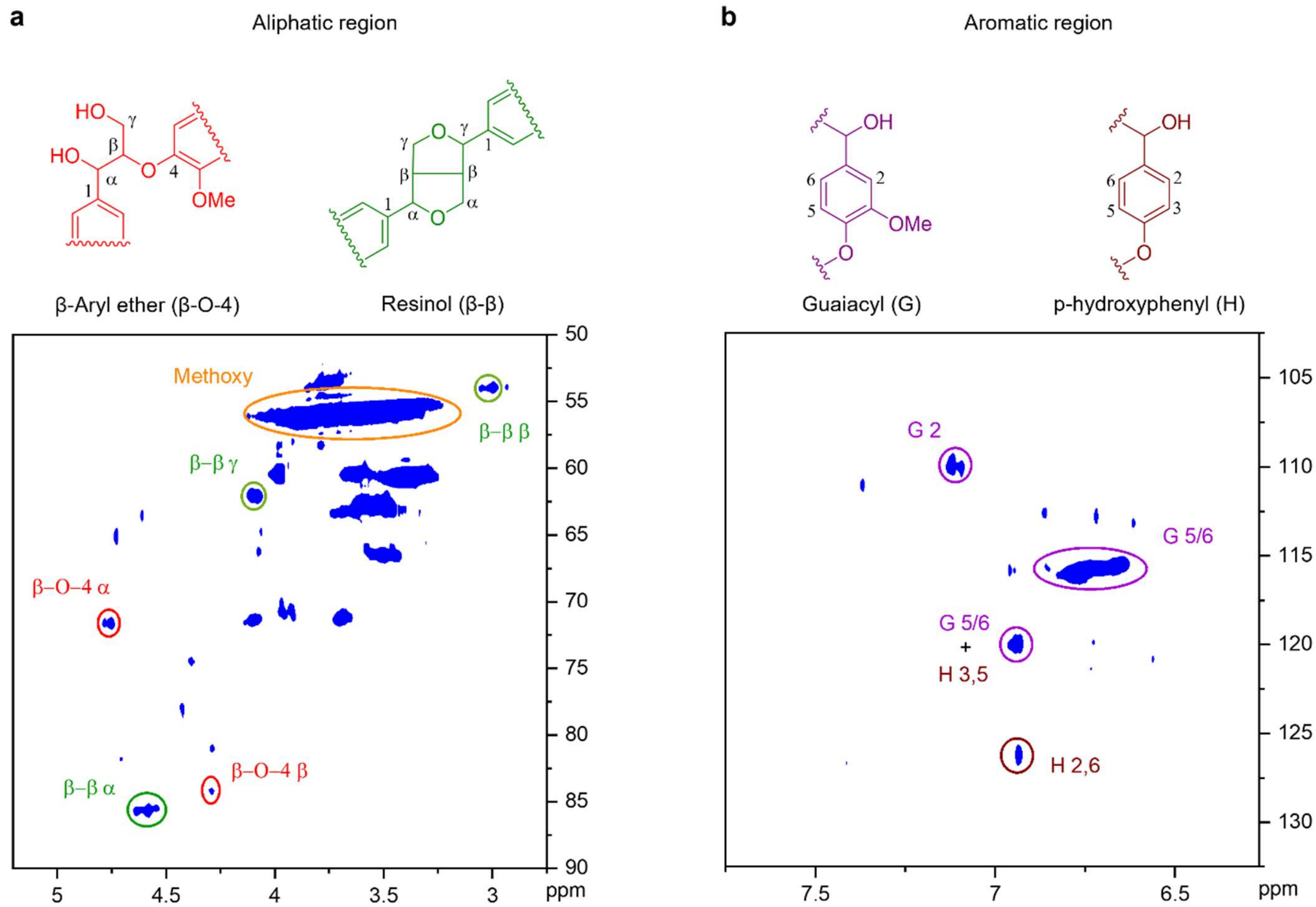

Figure S11: 2D HSQC NMR spectrum of Kraft lignin upon saturation in EtOAc with selected structural features assigned in the (**a**) aliphatic and (**b**) aromatic region.

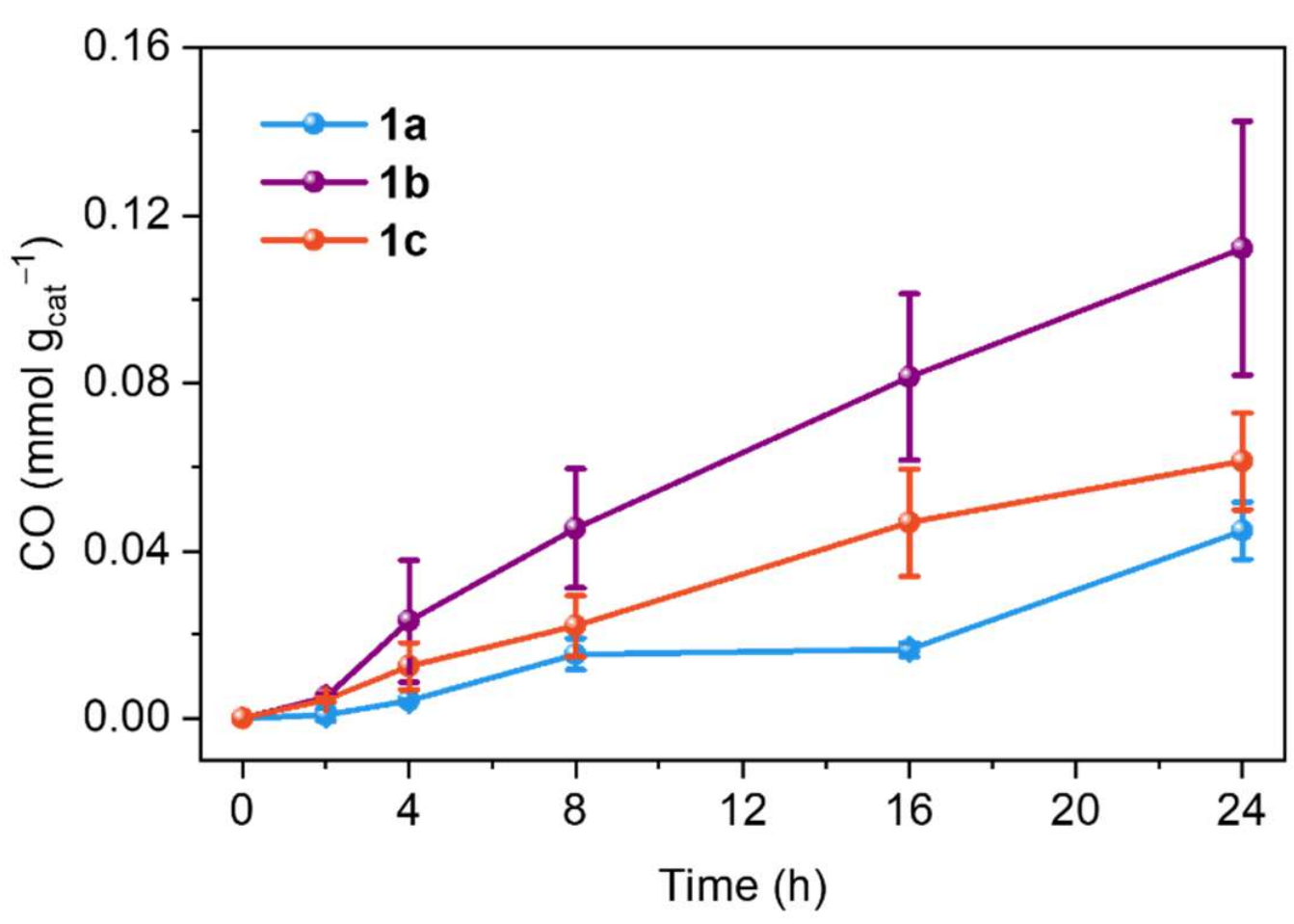

Figure S12: Photocatalytic CO generation (**b**) (447 nm, 120 mW cm^{-2}) over 24 h with CdS-AET QDs (1 µM) in 3 mL H_2O/MeCN (2:1, 25 °C, N_2 atmosphere) from 5 mM lignin models from 2-phenoxy-1-phenylethanol (PP-ol, **1a**), 1-(3,4-dimethoxyphenyl)-2-(2-methoxyphenoxy)-1,3-propanediol (**1b**) and guaiacyl glycerol-β-guaiacyl ether (**1c**).

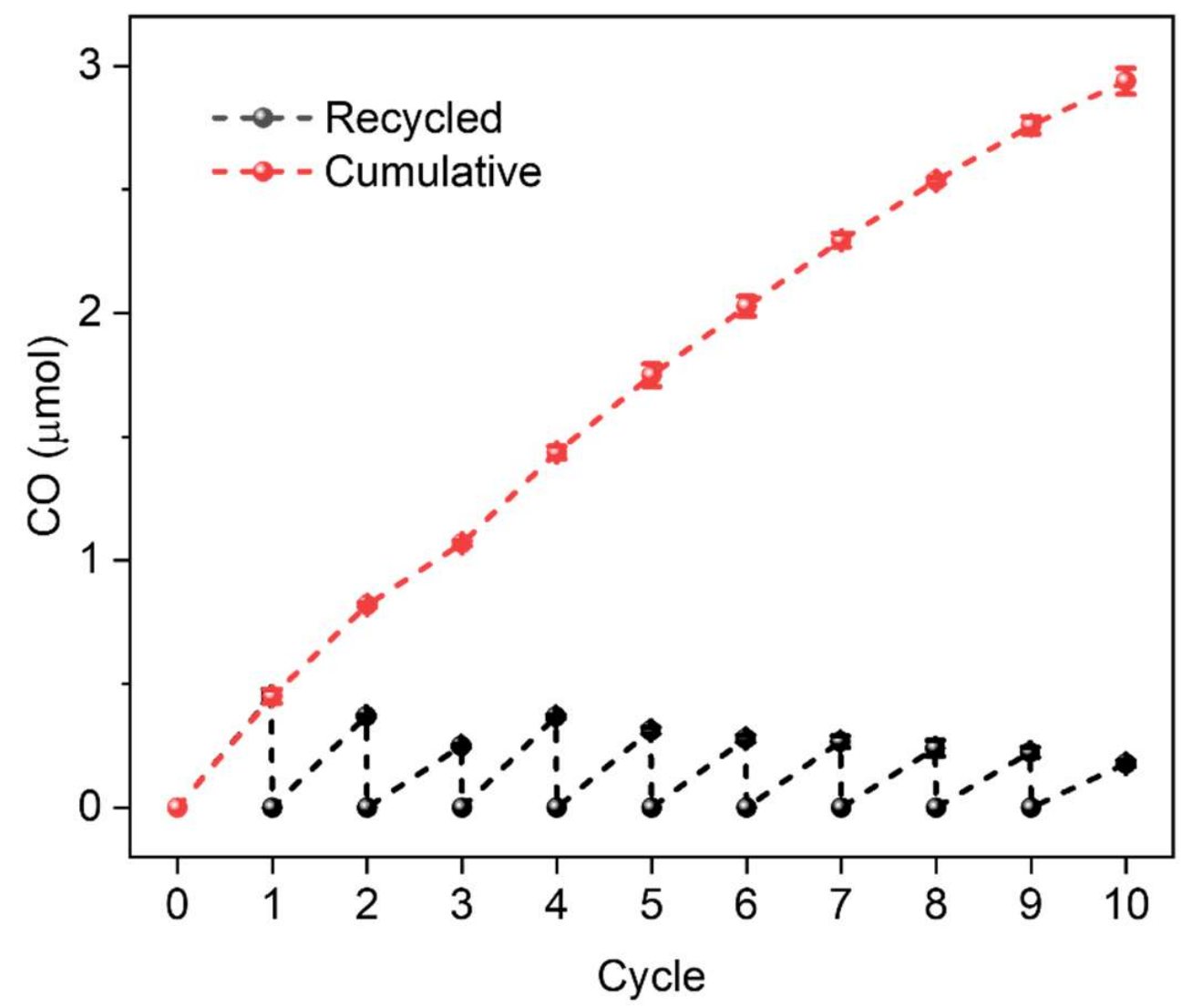

Figure S13: CO generation using 1 µM CdS-AET in H_2O and Kraft lignin saturated EtOAc (1:1, 3 mL). The recycling test was conducted by washing the aqueous phase after each run and adding fresh H_2O and EtOAc layer. 0.09 µmol additional AET ligand was added for each cycle.

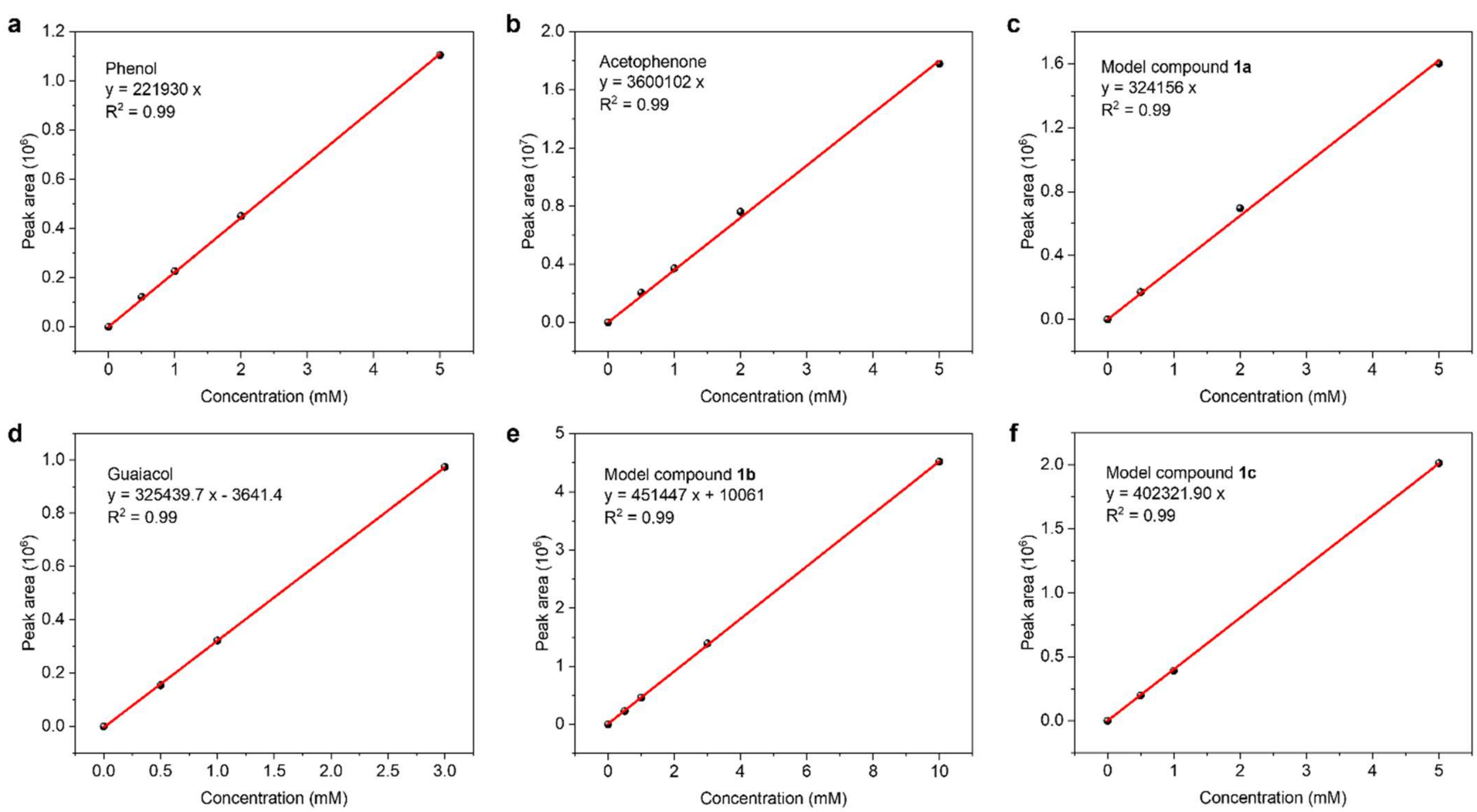

Figure S14: Calibration curves obtained over HPLC for the quantification of phenol (**a**), acetophenone (**b**), model compound 2-phenoxy-1-phenylethanol (**1a**) (**c**), guaiacol (**d**), and dilignol compound 1-(3,4-dimethoxyphenyl)-2-(2-methoxyphenoxy)-1,3-propanediol (**1b**) (**e**) and guaiacyl glycerol-β-guaiacyl ether (**1c**) (**f**).

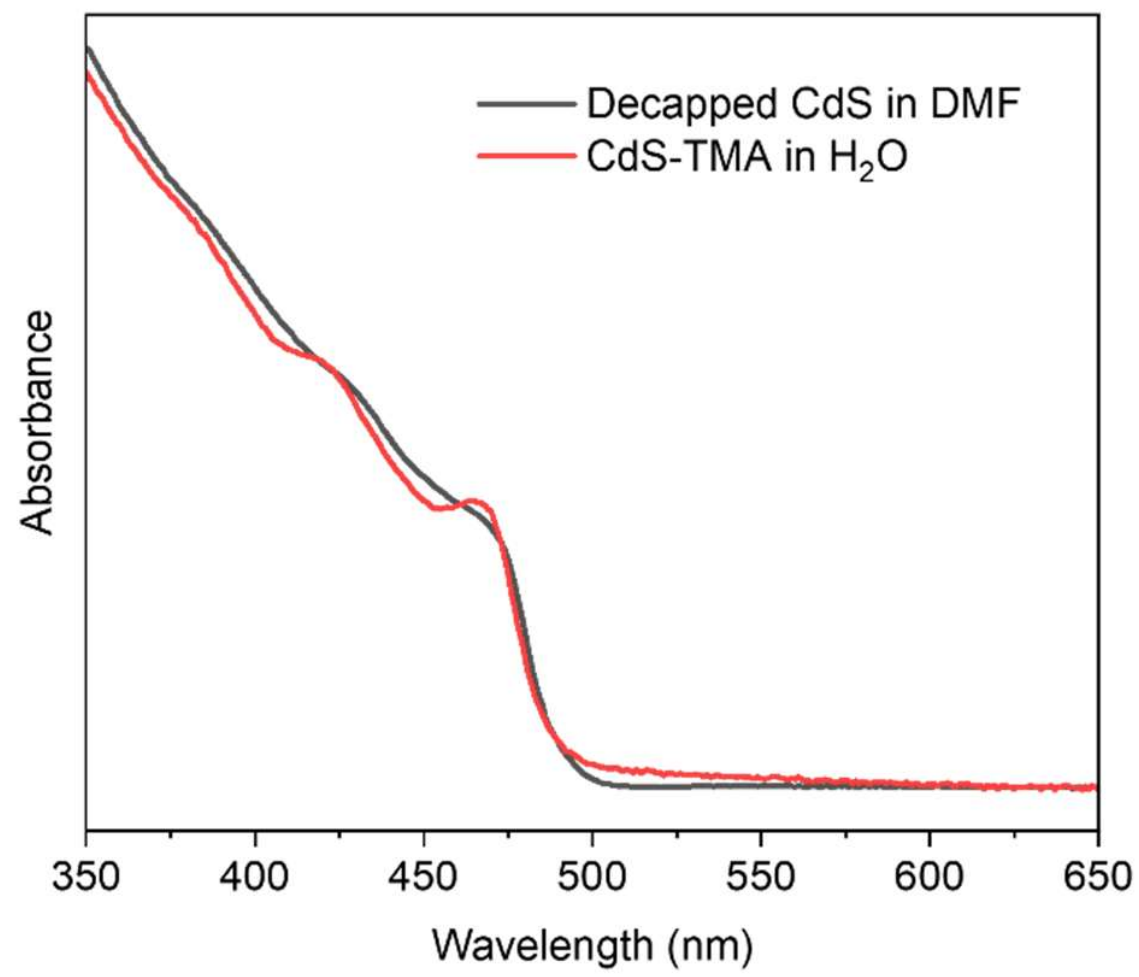

Figure S15: Normalized UV-Vis absorption spectra of decapped CdS QDs in DMF and CdS QDs capped with 2-mercaptoethyl-N,N,N-trimethylammonium chloride (TMA) ligand.

Supplementary References

(1) Huang, L.; Wang, X.; Yang, J.; Liu, G.; Han, J.; Li, C. Dual Cocatalysts Loaded Type I CdS/ZnS Core/Shell Nanocrystals as Effective and Stable Photocatalysts for H_2 Evolution. *J. Phys. Chem. C* **2013**, *117* (22), 11584–11591.

(2) Boles, M. A.; Ling, D.; Hyeon, T.; Talapin, D. V. The Surface Science of Nanocrystals. *Nat. Mater.* **2016**, *15* (2), 141–153.

(3) Yu, W. W.; Qu, L.; Guo, W.; Peng, X. Experimental Determination of the Extinction Coefficient of CdTe, CdSe, and CdS Nanocrystals. *Chem. Mater.* **2003**, *15* (14), 2854–2860.

(4) Moulder, J. F. *Handbook of X-Ray Photoelectron Spectroscopy: A Reference Book of Standard Spectra for Identification and Interpretation of XPS Data*; Physical Electronics Division, Perkin-Elmer Corporation, 1992.

(5) Chang, C. M.; Orchard, K. L.; Martindale, B. C. M.; Reisner, E. Ligand Removal from CdS Quantum Dots for Enhanced Photocatalytic H_2 Generation in pH Neutral Water. *J. Mater. Chem. A* **2016**, *4* (8), 2856–2862.

(6) Chalker, J. M.; Lercher, L.; Rose, N. R.; Schofield, C. J.; Davis, B. G. Conversion of Cysteine into Dehydroalanine Enables Access to Synthetic Histones Bearing Diverse Post-Translational Modifications. *Angew. Chem. Int. Ed.* **2012**, *51* (8), 1835–1839.

(7) Roure, B.; Alonso, M.; Lonardi, G.; Yildiz, D. B.; Buettner, C. S.; Dos Santos, T.; Xu, Y.; Bossart, M.; Derdau, V.; Méndez, M.; Llaveria, J.; Ruffoni, A.; Leonori, D. Photochemical Permutation of Thiazoles, Isothiazoles and Other Azoles. *Nature* **2025**, *637* (8047), 860–867.

(8) Gilman, H.; Catlin, W. E. N-Propylbenzene. *Org. Synth.* **1925**, *4*, 59.

(9) Lee, C. C.; Zohdi, H. F.; Sallam, M. M. M. Hydrogen-Deuterium Exchanges in a Friedel-Crafts Reaction. *J. Org. Chem.* **1985**, *50* (5), 705–707.

(10) Nakamura, K.; Nakajima, T.; Aoyama, T.; Okitsu, S.; Koyama, M. One-Pot Esterification and Amidation of Phenolic Acids. *Tetrahedron* **2014**, *70* (43), 8097–8107.

(11) Mitra, S.; Vahey, D. M.; Bonke, S. A.; Reisner, E. Compartmentalised Carbonylation Cascade Initiated by Photocatalytic CO_2 Reduction. *ChemRxiv* **2025** (1219).

(12) Mansfield, S. D.; Kim, H.; Lu, F.; Ralph, J. Whole Plant Cell Wall Characterization Using Solution-State 2D NMR. *Nat. Protoc.* **2012**, *7* (9), 1579–1589.

(13) Lancefield, C. S.; Ojo, O. S.; Tran, F.; Westwood, N. J. Isolation of Functionalized Phenolic Monomers through Selective Oxidation and C−O Bond Cleavage of the β-O-4 Linkages in Lignin. *Angew. Chem. Int. Ed.* **2015**, *54* (1), 258–262.

(14) Wu, X.; Fan, X.; Xie, S.; Lin, J.; Cheng, J.; Zhang, Q.; Chen, L.; Wang, Y. Solar Energy-Driven Lignin-First Approach to Full Utilization of Lignocellulosic Biomass under Mild Conditions. *Nat. Catal.* **2018**, *1* (10), 772–780.

(15) Chen, L.; Liu, Y.; Mitra, S.; Kim, D.; Huang, Z.; Vahey, D. M.; Bin Mohamad Annuar, A.; Reisner, E. Solar Lignin Reforming with Tunable Selectivity Using a Hybrid Photocatalyst in Aqueous Solution. *J. Am. Chem. Soc.* **2025**, *147* (47), 43509–43516.

(16) Xu, X.; Dai, S.; Xu, S.; Zhu, Q.; Li, Y. Efficient Photocatalytic Cleavage of Lignin Models by a Soluble Perylene Diimide/Carbon Nitride S-Scheme Heterojunction. *Angew. Chem. Int. Ed.* **2023**, *62* (44), e202309066.

(17) Liu, H.; Li, H.; Lu, J.; Zeng, S.; Wang, M.; Luo, N.; Xu, S.; Wang, F. Photocatalytic Cleavage of C–C Bond in Lignin Models under Visible Light on Mesoporous Graphitic Carbon Nitride through π–π Stacking Interaction. *ACS Catal.* **2018**, *8* (6), 4761–4771.

(18) Luo, N.; Wang, M.; Li, H.; Zhang, J.; Hou, T.; Chen, H.; Zhang, X.; Lu, J.; Wang, F. Visible-Light-Driven Self-Hydrogen Transfer Hydrogenolysis of Lignin Models and Extracts into Phenolic Products. *ACS Catal.* **2017**, *7* (7), 4571–4580.

(19) Han, G.; Yan, T.; Zhang, W.; Zhang, Y. C.; Lee, D. Y.; Cao, Z.; Sun, Y. Highly Selective Photocatalytic Valorization of Lignin Model Compounds Using Ultrathin Metal/CdS. *ACS Catal.* **2019**, *9* (12), 11341–11349.

(20) Wang, M.; Lu, J.; Li, L.; Li, H.; Liu, H.; Wang, F. Oxidative C(OH)C Bond Cleavage of Secondary Alcohols to Acids over a Copper Catalyst with Molecular Oxygen as the Oxidant. *J. Catal.* **2017**, *348*, 160–167.

(21) Wang, M.; Lu, J.; Zhang, X.; Li, L.; Li, H.; Luo, N.; Wang, F. Two-Step, Catalytic C–C Bond Oxidative Cleavage Process Converts Lignin Models and Extracts to Aromatic Acids. *ACS Catal.* **2016**, *6* (9), 6086–6090.

(22) Rinesch, T.; Mottweiler, J.; Puche, M.; Concepción, P.; Corma, A.; Bolm, C. Mechanistic Investigation of the Catalyzed Cleavage for the Lignin β-O-4 Linkage: Implications for Vanillin and Vanillic Acid Formation. *ACS Sustain. Chem. Eng.* **2017**, *5* (11), 9818–9825.
(23) vom Stein, T.; den Hartog, T.; Buendia, J.; Stoychev, S.; Mottweiler, J.; Bolm, C.; Klankermayer, J.; Leitner, W. Ruthenium-Catalyzed C−C Bond Cleavage in Lignin Model Substrates. *Angew. Chem., Int. Ed.* **2015**, *54* (20), 5959–5863.
